# Old Concepts, New Application – Additive‐Free Hydrogenation of Nitriles Catalyzed by an Air Stable Alkyl Mn(I) Complex

**DOI:** 10.1002/adsc.201901040

**Published:** 2019-10-28

**Authors:** Stefan Weber, Luis F. Veiros, Karl Kirchner

**Affiliations:** ^1^ Institute of Applied Synthetic Chemistry Vienna University of Technology Getreidemarkt 9/163-AC A-1060 Wien Austria; ^2^ Centro de Química Estrutural, Instituto Superior Técnico Universidade de Lisboa Av. Rovisco Pais No. 1 1049-001 Lisboa Portugal

**Keywords:** manganese, alkyl complexes, hydrogenation, nitriles, migratory insertion

## Abstract

An efficient additive‐free manganese‐catalyzed hydrogenation of nitriles to primary amines with molecular hydrogen is described. The pre‐catalyst, a well‐defined bench‐stable alkyl bisphosphine Mn(I) complex *fac*‐[Mn(dpre)(CO)_3_(CH_3_)] (dpre=1,2‐bis(di‐*n*‐propylphosphino)ethane), undergoes CO migratory insertion into the manganese‐alkyl bond to form acyl complexes which upon hydrogenolysis yields the active coordinatively unsaturated Mn(I) hydride catalyst [Mn(dpre)(CO)_2_(H)]. A range of aromatic and aliphatic nitriles were efficiently and selectively converted into primary amines in good to excellent yields. The hydrogenation of nitriles proceeds at 100 °C with a catalyst loading of 2 mol % and a hydrogen pressure of 50 bar. Mechanistic insights are provided by means of DFT calculations.

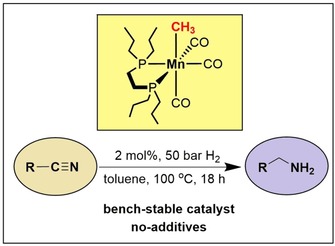

## Introduction

**Scheme 1:**
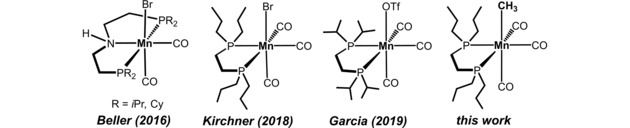
Manganese Catalysts for the Hydrogenation of Nitriles.

Amines constitute an important class of organic compounds which are used as versatile building blocks for the bulk and fine chemical industries.^1^ A very attractive, atom‐economic, and sustainable route to obtain selectively primary amines is the hydrogenation of nitriles with molecular hydrogen utilizing well‐defined catalysts based on Earth abundant non‐precious metals.^2^, ^3^, ^4^ In particular base metals such as iron and manganese are promising candidates as these belong to the most abundant metals in the Earth crust, are inexpensive, and exhibit a low environmental impact. While iron, cobalt, and nickel catalysts for the hydrogenation of polar multiple bonds such as carbonyl compounds, imines, and nitriles had been subject of intense investigation over the past decade,^5^, ^6^, ^7^, ^8^, ^9^, ^10^, ^11^ low valent Mn(I) catalysts just appeared in 2016 as new but very powerful players in this fast growing area.^6^, ^7^, ^8^, ^9^, ^10^, ^11^ As hydrogenation of nitriles is concerned, several well‐defined catalysts based on iron,^12^ cobalt^13^ and lately also manganese (Scheme 1)^14^ have been described.^15^ It is interesting to note that most of these reactions require additives (strong base) in order to activate the pre‐catalysts.

**Scheme 2:**
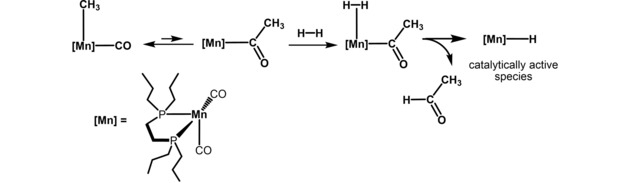
Formation of an Unsaturated Mn(I) Hydride Species *via* Alkyl Migration and Hydrogenolysis of an Acyl Intermediate.

In this article, we report on the synthesis and application of a bench‐stable and well‐defined alkyl bisphoshine Mn(I) complex as efficient pre‐catalyst for the selective hydrogenation of nitriles to give primary amines. It has to be noted that this is the first example of a manganese‐catalyzed hydrogenation of nitriles without the need of an additive (base) utilizing a well‐defined Mn(I) complex. The activation of the pre‐catalyst takes advantage of the fact that (i) CO ligands of Mn(I) alkyl carbonyl complexes are able to undergo migratory insertions to form acyl complexes in the presence of strong field ligands such as CO or tertiary phosphines – *a well‐known textbook reaction*.^16^ and that (ii) heterolytic cleavage of molecular hydrogen by acyl complexes affords aldehydes and coordinatively unsaturated metal hydride intermediates – a *key step in both stoichiometric and catalytic hydroformylations of alkenes*.^17^ Thus, the catalytic hydrogenation of nitriles is initiated by migratory insertion of a CO ligand into the Mn‐alkyl bond to yield acyl intermediates which undergo rapid hydrogenolysis to form the active 16e^−^ Mn(I) hydride catalysts as shown in Scheme 2.

## Results and Discussion

**Scheme 3:**
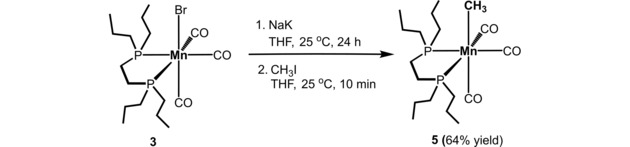
Synthesis of *fac‐*[Mn(dpre)(CO)_3_(CH_3_)] (**5**).

In addition to the known Mn(I) complexes [Mn(CO)_5_(CH_3_)] (**1**),^18^, ^19^ *fac*‐[Mn(bipy)(CO)_3_(CH_3_)] (**2**),^20^ the new Mn(I) complexes *fac‐*[Mn(dpre)(CO)_3_(H)] (dpre=1,2‐bis(di‐*n*‐propyl‐phosphino)ethane) (**4**) and *fac*‐[Mn(dpre)(CO)_3_(CH_3_)] (**5**)^21^ were synthesized, characterized, and applied. The hydride complex *fac*‐[Mn(dpre)(CO)_3_(H)] (**4**) was obtained by reacting a suspension of [Mn_2_(CO)_10_] and 1,2‐bis(dipropylphosphino)ethane in anhydrous *n*‐pentanol at 140 °C for 5 h in low yield (14%). The neutral methyl Mn(I) complex **5** was obtained by reacting *fac*‐[Mn(dpre)(CO)_3_(Br)] (**3**) with NaK and subsequently with CH_3_I in 64% isolated yield (Scheme 3). The resulting off‐white complex was fully characterized by ^1^H, ^13^C{^1^H} and ^31^P{^1^H} NMR and IR spectroscopy, and high‐resolution mass spectrometry. The IR spectrum contains strong CO stretching vibrations at 1974, 1888 and 1835 cm^−1^ which clearly indicate the coordination of three CO ligands to the metal center in a facial arrangement. In the ^1^H and ^13^C{^1^H} NMR spectra the methyl group gives rise to triplet resonances at −0.70 (*J*=9.0 Hz) and −16.8 ppm (*J*=18.2 Hz), respectively.

**Scheme 4:**
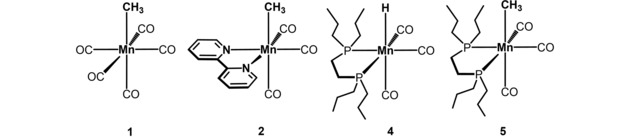
Mn(I) Complexes Tested as Catalysts.

The catalytic performance of complexes **1**, **2**, **4**, and **5** (Scheme 4) was then investigated for the hydrogenation of 4‐fluorobenzonitrile as model substrate. Selected optimization experiments are depicted in Table 1. With a catalyst loading of 3 mol% at 100 °C and a hydrogen pressure of 50 bar H_2_ in toluene for 18 h no reaction took place with complexes **1**, **2** and **4** as pre‐catalysts. This clearly emphasizes that strongly electron donating co‐ligands such as alkyl bisphosphines are required to achieve high catalytic activity. Moreover, the coordinatively saturated and inert hydride complex **4** is inactive due to the lack of a vacant coordination site. Accordingly, complex **4** is not an intermediate in the catalytic process which is obviously a metal‐centered, i. e., an inner‐sphere, reaction. Under the same reaction conditions with complex **5** but a catalyst loading of 2 mol%, 4‐fluorobenzylamine was obtained in 95% yield (Table 1, entry 4). Similar results were obtained with **5** in *i*PrOH (Table 1, entry 5), whereas no reaction took place in this solvent in the absence of H_2_ thus clearly excluding a transfer hydrogenation process (Table 1, entry 6). At lower temperature (80 °C) or a lower catalyst loading (1 mol%), the catalytic activity dropped significantly and no or lower conversions of 4‐fluorobenzonitrile were achieved (Table 1, entries 7 and 8).

**Table 1:** Optimization of the Reaction Conditions for the Hydrogenation of 4‐Fluorobenzonitrile.^[a]^

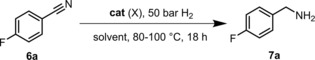						
entry	catalyst	X (mol%)	solvent	T (°C)	conversion (%)^[b]^	yield (%)^[c]^
1	[Mn(CO)_5_(CH_3_)] (**1**)	3	toluene	100	–	–
2	*fac*‐[Mn(bipy)(CO)_3_(CH_3_)] (**2**)	3	toluene	100	–	–
3	*fac*‐[Mn(dpre)(CO)_3_(H)] (**4**)	3	toluene	100	–	–
4	*fac*‐[Mn(dpre)(CO)_3_(CH_3_)] (**5**)	2	toluene	100	>99	95
5	*fac*‐[Mn(dpre)(CO)_3_(CH_3_)] (**5**)	2	*i*PrOH	100	>99	93
6^[d]^	*fac*‐[Mn(dpre)(CO)_3_(CH_3_)] (**5**)	2	*i*PrOH	100	–	–
7	*fac*‐[Mn(dpre)(CO)_3_(CH_3_)] (**5**)	1	toluene	100	37	11
8	*fac*‐[Mn(dpre)(CO)_3_(CH_3_)] (**5**)	2	toluene	80	–	–

^[a]^ Reaction conditions: 4‐Fluorobenzonitrile (0.6 mmol), 50 bar H_2_, 5 mL solvent, 18 h. ^[b]^ Conversion determined by ^19^F{^1^H} NMR analysis. ^[c]^ Yield determined by ^19^F{^1^H} NMR analysis using fluorobenzene as standard. ^[d]^ In the absence of H_2_.

Having established the best reaction conditions, the applicability of catalyst **5** is demonstrated in the selective hydrogenation of various nitriles, including substituted aromatic, benzylic, aliphatic nitriles and dinitriles. The results for aromatic substrates are shown in Tables 2 and 3. Substrates with electron‐withdrawing substituents such as halides containing substrate are very efficiently converted to amines in excellent yields (Table 2, entries 1–5). Good yields are achieved for substrates with electron‐donating substituents such as methyl, methoxy and amino groups (Table 2, entries 7–9). Heterocycles, for instance, pyridine and thiophene derivatives are well tolerated (Table 2, entries 10–12).

**Table 2:** Hydrogenation of Various (Hetero)aromatic Nitriles Catalyzed by **5**.^[a]^

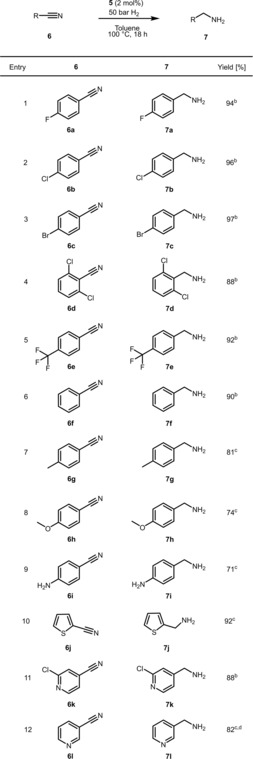

^[a]^ Reaction conditions: Substrate (0.6 mmol), catalyst **5** (2 mol%), 5 mL anhydrous toluene, 100 °C 50 bar H_2_, 18 h. ^[b]^ Isolated yields as ammonium hydrochloride. ^[c]^ GC yield. ^[d]^ 3 mol%. ^[e]^ 72 h. ^[f]^ 48 h

**Table 3:** Hydrogenation of Various Aliphatic Nitriles and Dinitriles Catalyzed by **5**.^[a]^

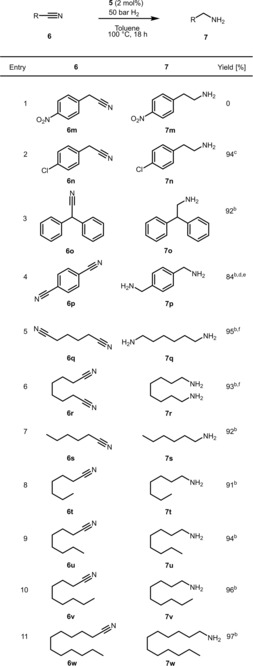

^[a]^ Reaction conditions: Substrate (0.6 mmol), catalyst **5** (2 mol%), 5 mL anhydrous toluene, 100 °C 50 bar H_2_, 18 h. ^[b]^ Isolated yields as ammonium hydrochloride. ^[c]^ GC yield. ^[d]^ 3 mol%. ^[e]^ 72 h. ^[f]^ 48 h.

No reaction was observed in the case of 2‐(4‐nitrophenyl)acetonitrile (Table 3, entry 1) indicating catalyst deactivation. Dinitriles were reduced to the corresponding diamines in good to excellent yields (Table 3, entries 4–6). Interestingly, aliphatic dinitriles, such as adiponitrile (Table 3, entry 5) gave higher yields in comparison to the aromatic analogues (Table 3, entry 4). Finally, linear aliphatic nitriles were reduced to the corresponding amines in excellent yields (Table 3, entries 7–11).

**Figure 1:**
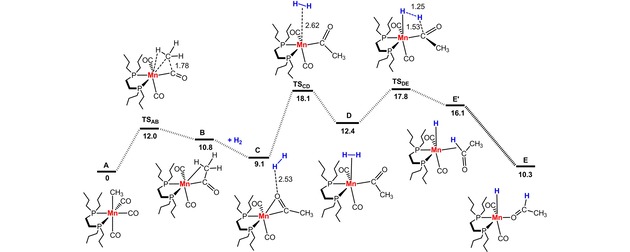
Free Energy Profile Calculated for the Formation and Hydrogenolysis an Acyl Intermediate. Free Energies (kcal/mol) are Referred to *fac*‐[Mn(dpre)(CO)_3_(CH_3_)] (**5**) (**A** in the calculations).

**Figure 2:**
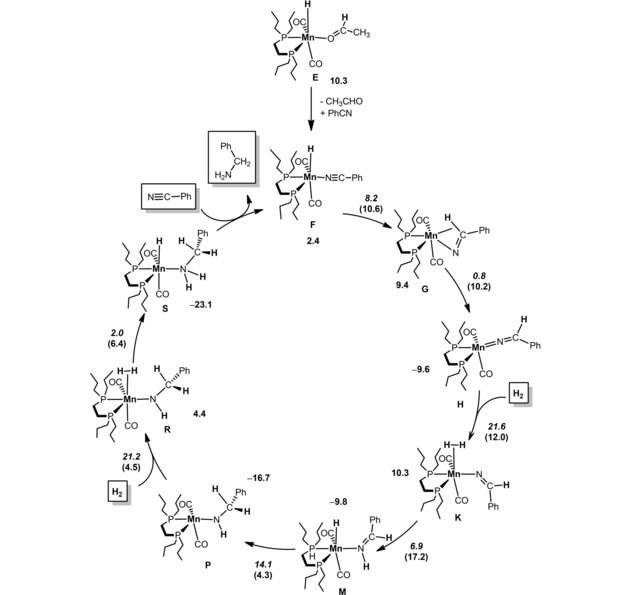
Simplified Catalytic Cycle for the Hydrogenation of Benzonitrile (Free energies in kcal/mol are referred to *fac*‐[Mn(dpre)(CO)_3_(CH_3_)] (**5**), barriers in italic and transition state energies in parenthesis).

A reasonable mechanism for the hydrogenation of nitriles was established by means of DFT calculations in toluene with benzonitrile as model substrate.^22^ The initiation step and the key intermediates along the catalytic cycle are presented in Figures 1 and 2 (see supporting information for details). The active species is formed by methyl migration to a CO ligand in complex **5** (**A** in the calculations, Figure 1) producing acyl complex **B** which is stabilized by a C−H agostic interaction. This step is endergonic (Δ*G*=10.8 kcal/mol) with an accessible barrier of 12.0 kcal/mol. Upon addition of H_2_ the dihydrogen complex **D** is formed. This step has a barrier of 9.0 kcal/mol and is slightly endergonic (Δ*G*=3.3 kcal/mol). Protonation of the acyl ligand through H‐transfer from the dihydrogen ligand takes place yielding finally intermediate **E**, featuring a hydride ligand and an O‐coordinated acetaldehyde. The last step, from **D** to **E**, goes over a 5.4 kcal/mol barrier and has a favorable free energy balance of −2.1 kcal/mol. The highest barrier along the initiation process is 18.1 kcal/mol corresponding to the energy of transition state **TS_CD_**.

A simplified catalytic cycle is depicted in Figure 2. The reaction begins with exchange of the aldehyde in intermediate **E** by one molecule of nitrile to form **F**, the initial species in the cycle with an N‐coordinated benzonitrile. From here the reaction consists of two consecutive H_2_ additions to the substrate going from a nitrile to an amine group. The catalytic cycle starts with hydride transfer to the C‐atom of the nitrile group producing an intermediate with a side‐bonded C=N double bond that is further stabilized by a C−H agostic interaction (**G**). This is an endergonic step (Δ*G*=7.0 kcal/mol) with a moderate barrier of 8.2 kcal/mol. A ligand rearrangement from side to end on coordination leads to intermediate **H**. Here, the PhCHC=N ligand is coordinated by the N‐atom becoming a 4‐electron donor and establishing a Mn=N double bond as indicated by a short Mn−N distance (1.76 Å) and a Mn−N−C angle of 173° approaching linearity. The free energy balance for that rearrangement is very favorable: Δ*G*=−19.0 kcal/mol. The path proceeds with H_2_ coordination reaching intermediate **K**, a dihydrogen species. Overall, the coordination of dihydrogen overcomes a significant barrier of 21.6 kcal/mol, being also unfavorable, from the thermodynamic point of view, **K** being 19.9 kcal/mol less stable than **H**. The reaction continues with N‐protonation *via* H‐transfer from the dihydrogen ligand. This leads to intermediate **M** presenting an N‐coordinated imine ligand, with a barrier of 6.9 kcal/mol and a favorable free energy balance of Δ*G*=−20.1 kcal/mol. This concludes the first part of the mechanism, where one equivalent of H_2_ was added to the initial benzonitrile forming the corresponding imine PhCH=NH.

The second part of the catalytic cycle starts with a rearrangement of the coordination mode of the imine ligand in **M**, followed by hydride transfer to the imine C‐atom resulting in intermediate **P** containing a N‐coordinated amido ligand. The overall process has a barrier of 14.1 kcal/mol and is favorable by −6.9 kcal/mol.^23^ The mechanism proceeds with coordination of the second H_2_ molecule resulting in the dihydrogen complex **R** which features an amido co‐ligand. Coordination of the second dihydrogen molecule is equivalent to what was found for the first one with similar barrier and free energy balance (21.2 kcal/mol).

The final step along the path corresponds to protonation of the N‐atom in **R**, in a facile step with a negligible barrier of 2.0 kcal/mol and a very favorable free energy balance (Δ*G*=−27.5 kcal/mol). In the final intermediate, **S**, the reaction product (benzylamine) is already formed and remains N‐coordinated to the metal. From **S**, ligand exchange with loss of the product and addition of a new nitrile molecule closes the cycle and regenerates the starting intermediate **F** with a neutral free energy balance of Δ*G*=0 kcal/mol. The overall reaction barrier is 26.8 kcal/mol, measured from **H** to the transition state of N‐protonation (**TS_KL_**, see supporting information). This is a high barrier that may be slightly overestimated but reflects the reaction conditions (18 h at 100 °C).

## Conclusion

In sum, we report an efficient hydrogenation of nitriles with molecular hydrogen catalyzed by a well‐defined, bench‐stable bisphosphine Mn(I) complex. Our results indicate that an inner‐sphere (metal‐centered) process without ligand participation is taking place. To the best of our knowledge, this is the first example of an additive‐free hydrogenation of nitriles catalyzed by a well‐defined Mn(I) complex. These reactions are atom economic implementing an inexpensive, earth abundant non‐precious metal catalyst. The catalytic process is initiated by migratory insertion of a CO ligand into the Mn‐alkyl bond to yield an acyl intermediate which undergoes rapid hydrogenolysis to form the active 16e^−^ Mn(I) hydride catalyst [Mn(dpre)(CO)_2_(H)] – a conceptually new approach in Mn(I) hydrogenation chemistry. A range of (hetero)aromatic and aliphatic nitriles were efficiently converted into primary amines, respectively, in good to excellent isolated yields. The hydrogenation of nitriles proceeds at 100 °C with a catalyst loading of 2 mol %. A hydrogen pressure of 50 bar was applied and the reaction time was 18 h. The mechanism obtained by means of DFT calculations consists of two successive and equivalent H_2_ additions to the initial nitrile substrate. The crucial features in the path are hydride transfer to the substrate C‐atom, followed by protonation of the corresponding N‐atom, deriving from key hydride and dihydrogen intermediates, respectively.

## Experimental Section

**General Information**. All reactions were performed under an inert atmosphere of argon by using Schlenk techniques or in a MBraun inert‐gas glovebox. The solvents were purified according to standard procedures. The deuterated solvents were purchased from Aldrich and dried over 3 Å molecular sieves. Complexes [Mn(CO)_5_(CH_3_)] (**1**),^24^ *fac*‐[Mn(bipy)(CO)_3_(CH_3_)] (**2**),^25^ *fac‐*[Mn(dpre)(CO)_3_(Br)] (dpre=1,2‐bis(di‐*n*‐propyl‐phosphino)ethane) (**3**),14b were synthesized according to literature.^1^H and ^13^C{^1^H}, and ^31^P{^1^H} NMR spectra were recorded on Bruker AVANCE‐250, AVANCE‐400, and AVANCE‐600 spectrometers. ^1^H and ^13^C{^1^H} NMR spectra were referenced internally to residual protio‐solvent, and solvent resonances, respectively, and are reported relative to tetramethylsilane (δ=0 ppm). ^31^P{^1^H} NMR spectra were referenced externally to H_3_PO_4_ (85%) (δ=0 ppm). Hydrogenation reactions were carried out in a Roth steel autoclave using a Tecsis manometer.

High resolution‐accurate mass data mass spectra were recorded on a hybrid Maxis Qq‐aoTOF mass spectrometer (Bruker Daltonics, Bremen, Germany) fitted with an ESI‐source. Measured accurate mass data of the [M]^+^ ions for confirming calculated elemental compositions were typically within ±5 ppm accuracy. The mass calibration was done with a commercial mixture of perfluorinated trialkyl‐triazines (ES Tuning Mix, Agilent Technologies, Santa Clara, CA, USA).

GC‐MS analysis was conducted on a ISQ LT Single quadrupole MS (Thermo Fisher) directly interfaced to a TRACE 1300 Gas Chromatographic systems (Thermo Fisher), using a Rxi‐5Sil MS (30 m, 0.25 mm ID) cross‐bonded dimethyl polysiloxane capillary column.

**Synthesis**. ***fac*** **‐[Mn(dpre)(CO)_3_(H)] (4)**. A microwave vial was charged with [Mn_2_(CO)_10_] (300 mg, 0.77 mmol), 1,2‐bis(dipropylphosphino)ethane (404 mg, 1.54 mmol) and anhydrous *n*‐pentanol (350 μL). The mixture was heated to 140 °C for 5 h. All volatiles were removed under vacuum affording a red oil. Anhydrous MeOH (3 mL) was added and the yellow solution was placed in the freezer giving a yellow solution and a colorless precipitate. The suspension was centrifuged and the mother liquor was decanted. The solid was washed three times with MeOH (1 mL) and the solid was dried under vacuum. Yield: 80 mg (14%) colorless solid. ^1^H NMR (δ, 400 MHz, C_6_D_6_, 20 °C): 1.56–0.85 (*m*, 25H), 0.76 (*t, J*=6.9 Hz, 6H), 0.70 (*t*, *J*=6.0 Hz, 6H), −9.25 (*t*, *J*=48.6 Hz, 1H). ^13^C{^1^H} NMR (δ, 101 MHz, C_6_D_6_, 20 °C): 224.6, 221.2, 32.6 (*vm*), 25.1 (*vt*, J=13.1 Hz), 16.8 (*vd*, J=6.1 Hz), 15.0 (*vm*). ^31^P{^1^H} NMR (δ, 162 MHz, C_6_D_6_, 20 °C): 86.8 (*s*). ATR‐IR (solid, cm^−1^): 1976 (*ν*_CO_), 1873 (*ν*_CO_), 1737 (*ν*_MnH_). HRMS (TOF ESI+): *m/z* calculated for C_17_H_31_MnO_3_P_2_ [M−H]^+^: 401.1202, found 401.1196.

***fac‐*** **[Mn(dpre)(CO)_3_(CH_3_)] (5)**. To a solution of *fac‐*[Mn(dpre)(CO)_3_(Br)] (**3**) (500 mg, 1.11 mmol) in anhydrous THF (30 mL) liquid Na/K alloy (2:3,110 mg, 3.33 mmol) was added and stirred for 24 h. The solution was filtrated *via* a syringe filter and methyl iodide (1 mL) was added and the mixture was stirred for 10 min. The solvent was removed under vacuum and the residue was extracted with CH_2_Cl_2_ (15 mL) giving an orange solution. Upon removal of the solvent a yellow oily residue was obtained which was dissolved in boiling anhydrous MeOH (10 mL) and the flask was stored in the freezer resulting in the crystallization of an off‐white solid. Yield: 293 mg (64%) off‐white solid. ^1^H NMR (δ, 400 MHz, C_6_D_6_, 20 °C): 1.72–1.33 (*m*, 14H), 1.33–1.19 (*m*, 3H), 1.19–0.93 (*m*, 7H), 0.87 (*t*, *J*=6.9 Hz, 6H), 0.80 (*t*, *J*=6.2 Hz, 7H), −0.70 (*t*, *J*=9.0 Hz, 3H). ^13^C{^1^H} NMR (δ, 101 MHz, C_6_D_6_, 20 °C): 31.4 (*vt*, J=12.1 Hz), 24.6 (*vd*, J=10.1 Hz), 23.9 (*vt*, J=20.2 Hz), 17.3 (*vd*, J=64.6 Hz), 15.7 (*vd*, J=12.1 Hz), −16.8 (*vt*, J=18.2 Hz), (CO not observed). ^31^P{^1^H} NMR (δ, 162 MHz, C_6_D_6_, 20 °C): 78.7 (*s*). ATR‐IR (solid, cm^−1^): 1974 (*ν*_CO_), 1888 (*ν*_CO_), 1853 (*ν*_CO_). HRMS (TOF ESI+): *m/z* calculated for C_17_H_31_MnO_3_P_2_ [M−CH_3_]^+^: 401.1202, found 401.1199.

**General procedure for the hydrogenation of nitriles**. Inside an Ar‐flushed glovebox, the nitrile (0.6 mmol, 1 equiv.) and *fac‐*[Mn(dpre)(CO)_3_(CH_3_)] (**5**) (0.006–0.022 mmol, 0.01–0.04 equiv.) were mixed with 5 mL of the anhydrous solvent and transferred into a steel autoclave, which was three times evacuated and flushed with Ar prior use. The autoclave was flushed three times with hydrogen, 50 bar H_2_ pressure were applied and the autoclave was placed in an oil bath (80–100 °C) and the reaction was run for 18 h. The autoclave was then cooled in an ice bath for 10 min. The reaction mixture was filtrated through a PTFE membrane and analyzed by ^19^F{^1^H} NMR for 4‐fluorobenzonitrile as test substrate using fluorobenzene as external standard.

**Isolation of product as ammonium salt**. The reaction mixture was filtrated through a short plug of Celite and diluted with Et_2_O to 25 mL and 1 M HCl in Et_2_O was added drop wise until precipitation was complete. The solution was decanted and the ammonium salt was several times washed with Et_2_O and dried under vacuum.

**Computational details**. The computational results presented have been achieved in part using the Vienna Scientific Cluster (VSC). All calculations were performed using the gaussian 09 software^26^ without symmetry constraints. The optimized geometries were obtained with the the PBE0 functional. That functional uses a hybrid generalized gradient approximation (GGA), including 25% mixture of Hartree‐Fock^27^ exchange with DFT^28^ exchange‐correlation, given by Perdew, Burke and Ernzerhof functional (PBE).^29^ The basis set used for the geometry optimizations (basis b1) consisted of the Stuttgart/Dresden ECP (SDD) basis set^30^ to describe the electrons of manganese, and a standard 6–31G(d,p) basis set^31^ for all other atoms. Transition state optimizations were performed with the Synchronous Transit‐Guided Quasi‐Newton Method (STQN) developed by Schlegel *et al*,^32^ following extensive searches of the Potential Energy Surface. Frequency calculations were performed to confirm the nature of the stationary points, yielding one imaginary frequency for the transition states and none for the minima. Each transition state was further confirmed by following its vibrational mode downhill on both sides and obtaining the minima presented on the energy profiles. The electronic energies (*E*_b1_) obtained at the PBE0/b1 level of theory were converted to free energy at 298.15 K and 1 atm (*G*_b1_) by using zero point energy and thermal energy corrections based on structural and vibration frequency data calculated at the same level. The free energy values presented were corrected for dispersion by means of Grimme DFT−D3 method^33^ with Becke and Johnson short distance damping.^34^ Solvent effects (toluene) were considered in all calculations using the Polarizable Continuum Model (PCM) initially devised by Tomasi and coworkers^35^ with radii and non‐electrostatic terms of the SMD solvation model, developed by Truhlar *et al*.^36^

## Supporting information

As a service to our authors and readers, this journal provides supporting information supplied by the authors. Such materials are peer reviewed and may be re‐organized for online delivery, but are not copy‐edited or typeset. Technical support issues arising from supporting information (other than missing files) should be addressed to the authors.

Supplementary
