# Supplementary material for: Old Concepts, New Application – Additive‐Free Hydrogenation of Nitriles Catalyzed by an Air Stable Alkyl Mn(I) Complex
Source: Adv Synth Catal. 2019 Oct 28;361(23):5412–20. doi: 10.1002/adsc.201901040 (PMC6916632; doi:10.1002/adsc.201901040)
Supplement: Supplementary file 1 — Supplementary [file ADSC-361-5412-s001.pdf]

Supporting Information

© Copyright Wiley-VCH Verlag GmbH & Co. KGaA, 69451 Weinheim, 2019

Old Concepts, New Application – Additive-Free Hydrogenation of Nitriles Catalyzed by an Air Stable Alkyl Mn(I) Complex

Stefan Weber, Luis F. Veiros, and Karl Kirchner*© 2019 The Authors. Published by Wiley-VCH Verlag GmbH & Co. KGaA.
This is an open access article under the terms of the Creative Commons Attribution License, which permits use, distribution and reproduction in any medium, provided the original work is properly cited.

Old Concepts, New Application - Additive-free Hydrogenation of Nitriles Catalyzed by an Air Stable Alkyl Mn(I) Complex

Stefan Weber,[a] Luis F. Veiros,[b] and Karl Kirchner*,[a]

[a] Institute of Applied Synthetic Chemistry, Vienna University of Technology, Getreidemarkt 9/163-AC, A-1060 Wien, Austria. E-mail: karl.kirchner@tuwien.ac.at

[b] Centro de Química Estrutural, Instituto Superior Técnico, Universidade de Lisboa, Av. Rovisco Pais No. 1, 1049-001 Lisboa, Portugal.

Supporting Information

1. Characterization of Organic Products **S2**

2. Tests for Homogeneous Catalysis **S4**

3. Additional Computational Details **S4**

4. References **S5**

5. NMR Spectra of All Complexes and Organic Products **S6**

1. Characterization of organic products

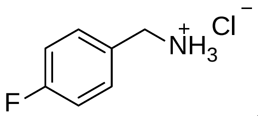

4-Flurobenzylammonium chloride[1] (**7a**): pale yellow solid (91 mg), ^{1}H NMR (δ, 400 MHz, D_2O, 20 ^{o}C): 7.37 – 7.30 (*m*, 2H), 7.04 (*m*, 2H), 4.03 (*s*, 2H). ^{13}C{^{1}H} NMR (δ, 101 MHz, D_2O, 20 ^{o}C):162.4, 131.0 , 128.6 , 115.8 , 42.4 .

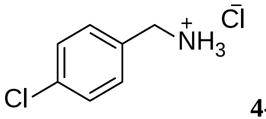

4-Chlorobenzylammonium chloride[2] (**7b**): white solid (102 mg), ^{1}H NMR (δ, 400 MHz, D_2O, 20 ^{o}C): 7.31 (*d*, *J* = 8.5 Hz, 2H), 7.24 (*d*, *J* = 8.4 Hz, 2H), 3.68 (*s*, 2H). ^{13}C{^{1}H} NMR (δ, 101 MHz, D_2O, 20 ^{o}C): 140.6 , 131.9 , 128.91 , 128.5 , 44.0 .

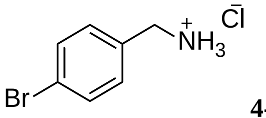

4-Bromobenzylammonium chloride[2] (**7c**): white solid (130 mg), ^{1}H NMR (δ, 400 MHz, D_2O, 20 ^{o}C): 7.54 (*d*, J = 8.1 Hz, 2H), 7.27 (*d*, J = 8.1 Hz, 2H), 4.06 (*s*, 2H). ^{13}C{^{1}H} NMR (δ, 101 MHz, D_2O, 20 ^{o}C):132.1 , 131.6 , 130.7 , 122.7 , 42.4 .

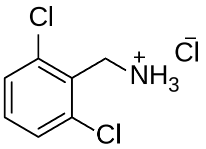

2,6-Dichlorobenzylammonium chloride[2] (**7d**): white solid (112 mg), ^{1}H NMR (δ, 400 MHz, D_2O, 20 ^{o}C): 7.44 (*d*, *J* = 7.6 Hz, 2H), 7.34 (*dd*, *J* = 9.0, 7.2 Hz, 1H), 4.46 (*s*, 2H). ^{13}C{^{1}H} NMR (δ, 101 MHz, D_2O, 20 ^{o}C): 135.9 , 131.8 , 128.8 , 128.20, 38.3.

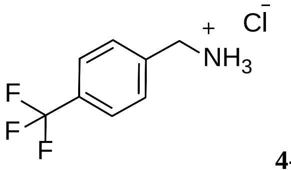

4-(Trifluoromethyl)benzylammonium chloride [1] (**7e**): white solid (114 mg), ^{1}H NMR (δ, 250 MHz, D_2O, 20 ^{o}C): 7.68 (*d*, J = 8.1 Hz, 2H), 7.50 (*d*, J = 8.1 Hz, 2H), 4.16 (*s*, 2H). ^{13}C{^{1}H} NMR (δ, 63 MHz, D_2O, 20 ^{o}C):136.5 , 130.3 , 139.2 , 126.0 (vq, J= , 42.9 .

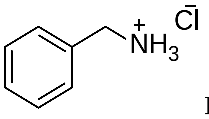

Benzylammonium chloride[2] (**7f**): white solid (75 mg), ^{1}H NMR (δ, 400 MHz, CD_3OD, 20 ^{o}C): 7.33 – 7.18 (*m*, 5H), 3.79 (*s*, 2H). ^{13}C{^{1}H} NMR (δ, 101 MHz, CD_3OD, 20 ^{o}C):142.3, 128.1 , 127.0 , 126.5, 45.3.

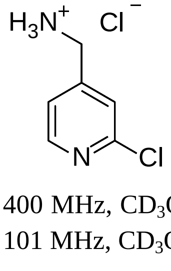

(2-chloropyridin-4-yl)methanammonium chloride[4] (**7k**): yellow solid (95 mg), ^{1}H NMR (δ, 400 MHz, CD_3OD, 20 ^{o}C): 8.47 (*d*, J = 23.0 Hz, 1H), 7.63 (*s*, 1H), 7.51 (*s*, 1H), 4.23 (*s*, 2H). ^{13}C{^{1}H} NMR (δ, 101 MHz, CD_3OD, 20 ^{o}C): 151.3 , 149.7 , 146.4 , 124.0 , 122.4 , 41.1 .

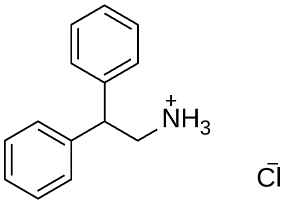
2,2 Diphenylethylammonium chloride[2] (**7o**): white solid (129 mg) ^{1}H NMR (δ, 400 MHz, D_2O, 20 ^{o}C): 7.33 (*d*, *J* = 4.4 Hz, 8H), 7.33 – 7.21 (*m*, 2H), 4.28 (*t*, *J* = 8.3 Hz, 1H), 3.66 (*d*, *J* = 8.3 Hz, 2H). ^{13}C{^{1}H} NMR (δ, 101 MHz, D_2O, 20 ^{o}C): 140.0, 129.3, 127.8, 127.7 , 48.6 , 43.1.

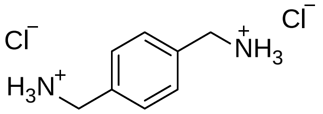
1,4-Phenylenedimethanammonium dichloride[1] (**7p**): yellow solid (105 mg) ^{1}H NMR (δ, 400 MHz, D_2O, 20 ^{o}C): 7.84 (*d*, J = 8.3 Hz, 2H), 7.54 (*d*, J = 8.2 Hz, 2H), 4.13 (*s*, 4H). ^{13}C{^{1}H} NMR (δ, 101 MHz, D_2O, 20 ^{o}C): 139.5, 135.9 , 133.5 , 130.6 , 42.7 .

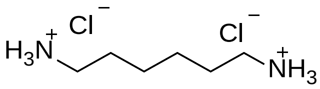
1,6-Hexandiammonium dichloride[1] (**7q**): white solid (105 mg), ^{1}H NMR (δ, 400 MHz, D_2O, 20 ^{o}C): 2.93 (*t*, J = 7.6 Hz, 4H), 1.61 (*quin*, J = 7.4 Hz, 4H), 1.40 – 1.29 (*m*, 4H). ^{13}C{^{1}H} NMR (δ, 101 MHz, D_2O, 20 ^{o}C): 39.4 , 26.5 , 25.1 .

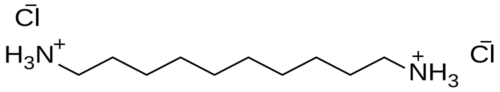
1,10-Decandiammonium dichloride[2] (**7r**): white solid (140 mg), ^{1}H NMR (δ, 400 MHz, D_2O, 20 ^{o}C): 2.88 (*t*, J = 7.4 Hz, 4H), 2.37 (*quin*, J = 6.8 Hz, 4H), 1.63 – 1.46 (*m*, 8H), 1.27 (*m*, 20H). ^{13}C{^{1}H} NMR (δ, 101 MHz, D_2O, 20 ^{o}C): 39.5, 28.17, 3.82 , 27.7, 25.5 , 16.3.

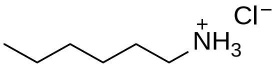
1-Hexanammonium chloride[1] (**7s**): white solid (106 mg) ^{1}H NMR (δ, 400 MHz, CD_3OD, 20 ^{o}C): 2.65 (*t*, J = 7.5 Hz, 2H), 1.53-1.47 (*m*, 2H), 1.44 – 1.22 (*m*, 6H), 0.95 (*t*, J = 6.9 Hz, 3H). ^{13}C{^{1}H} NMR (δ, 101 MHz, CD_3OD, 20 ^{o}C): 41.4 , 32.7 , 31.6 , 26.5 , 22.4 , 13.2 .

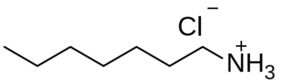
1-Heptanammonium chloride[3] (**7t**): white solid (83 mg), ^{1}H NMR (δ, 400 MHz, CD_3OD, 20 ^{o}C): 2.63 (*t*, *J* = 8.1 Hz, 3H), 1.48 (*quin*, *J* = 7.0 Hz, 2H), 1.41 – 1.25 (*m*, 8H), 0.93 (*t*, *J* = 6.7 Hz, 2H). ^{13}C{^{1}H} NMR (δ, 101 MHz, CD_3OD, 20 ^{o}C): 41.2 , 32.6 , 31.6 , 28.9 , 26.6 , 22.3 , 13.0 .

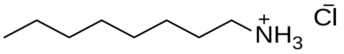
1-Octanammonium chloride[2] (**7u**): white solid (93 mg), ^{1}H NMR (δ, 400 MHz, CD_3OD, 20 ^{o}C): 2.91 (*t*, J = 7.6 Hz 2H), 1.66 (*quin*, J = 7.6 Hz, 2H), 1.46 – 1.20 (*m*, 10H), 0.89 (*t*, J = 6.8 Hz, 3H). ^{13}C{^{1}H} NMR (δ, 101 MHz, CD_3OD, 20 ^{o}C): 39.4 , 31.50, 28.8 , 27.2 , 26.09 , 22.3 , 13.0.

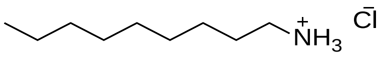
1-Nonanammonium chloride[2] (**7v**): white solid (103 mg), ^{1}H NMR (δ, 400 MHz, CD_3OD, 20 ^{o}C): 2.92 (*t*, J = 7.4 Hz 2H), 1.63 (*quin*, J = 7.5 Hz, 2H), 1.46 – 1.20 (*m*, 12H), 0.80 (*t*, J = 6.9 Hz, 3H). ^{13}C{^{1}H} NMR (δ, 101 MHz, CD_3OD, 20 ^{o}C): 39.6 , 31.6 , 29.1 , 28.1 , 28.7 , 26.9 , 26.1 , 22.4 , 13.6.

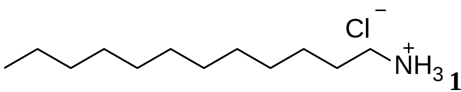
1-Dodecanammonium chloride[1] (**7v**): white solid (129 mg), ^{1}H NMR (δ, 400 MHz, CD_3OD, 20 ^{o}C): 2.63 (t, J = 7.3 Hz, 2H), 1.54 – 1.42 (m, 2H), 1.32 (m, 19H), 0.92 (t, J = 7.1 Hz, 3H). ^{13}C{^{1}H} NMR (δ, 101 MHz, CD_3OD, 20 ^{o}C): 41.3, 32.6, 31.7, 29.4, 26.7, 22.4, 13.1 .

2. Tests for Homogeneous Catalysis.

Mercury Drop Test: For this test, a drop of mercury was added to the pressure reaction vessel before the previously prepared reaction solution was injected (0.012 mmol, 2 mol% of **5**, 5.0 mL toluene). In comparison to the reaction in absence of mercury, no decrease of the catalytic activity and productivity took place.

3. Additional Computational Details

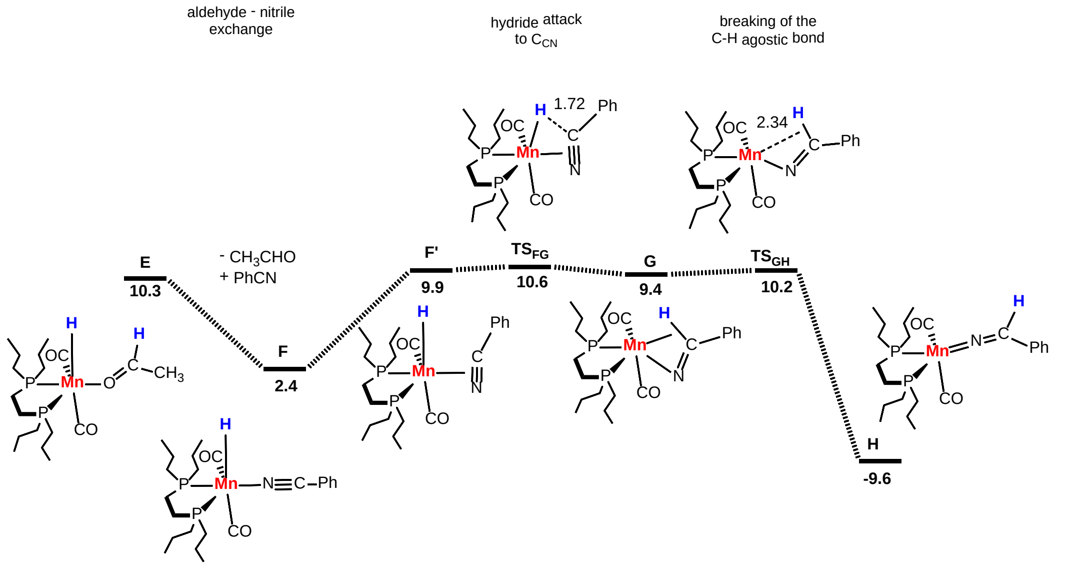

Figure S1. Free Energy profile for the catalytic reaction of nitrile hydrogenation (Free Energies (kcal/mol) are referred to *fac*-[Mn(dpre)(CO)$_3$(CH$_3$)] (**5**) (**A** in the calculations), distances in Å).

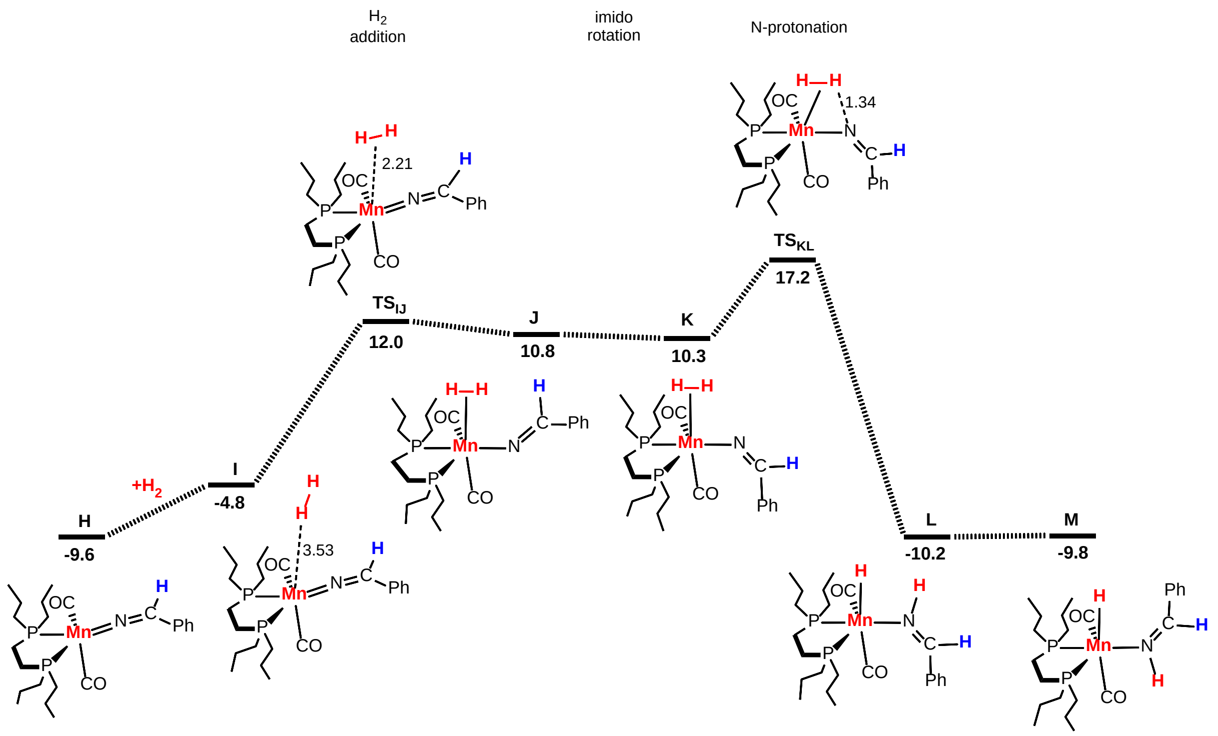

Figure S2. Free Energy profile for the catalytic reaction of nitrile hydrogenation (Free Energies (kcal/mol) are referred to *fac*-[Mn(dpre)(CO)$_3$(CH$_3$)] (**5**) (**A** in the calculations), distances in Å).

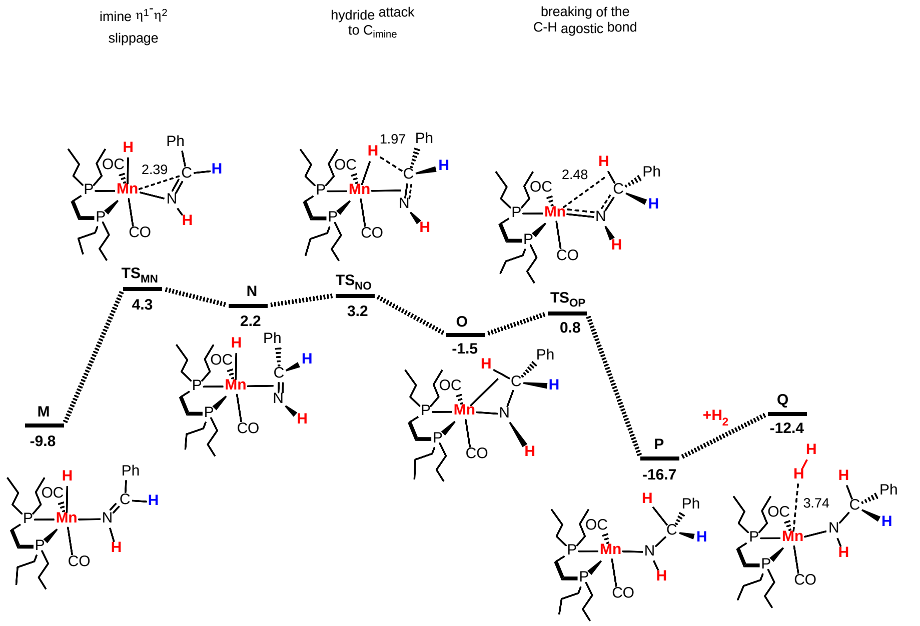

Figure S3. Free Energy profile for the catalytic reaction of nitrile hydrogenation (Free Energies (kcal/mol) are referred to *fac*-[Mn(dpre)$(CO)_3(CH_3)$] (**5**) (**A** in the calculations), distances in Å).

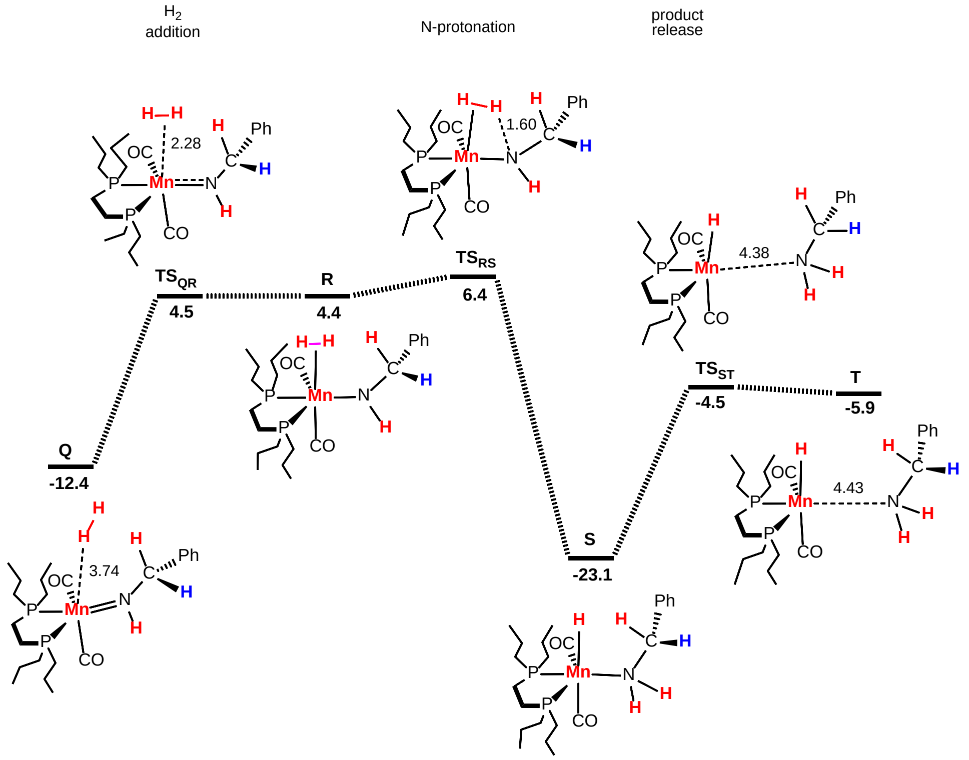

Figure S4. Free Energy profile for the catalytic reaction of nitrile hydrogenation (Free Energies (kcal/mol) are referred to *fac*-[Mn(dpre)$(CO)_3(CH_3)$] (**5**) (**A** in the calculations), distances in Å).

4. References

[1] C. Bornschein, S. Werkmeister, B. Wendt, H. Jiao, E. Alberico, W. Baumann, H. Junge, K. Junge, M. Beller *Nat. Commun.* **2014**, *5*, 4111.

[2] S. Weber, B. Stöger, K. Kirchner *Org. Lett.* **2018** *20*, 7212-7215.

[3] J. E. Torr, J. M. Large, E. McDonald *Comb. Chem. High Throughput Screen.* **2009**, *12*, 275-284.

[4] F. Corbellini, R. Fiammengo, P. Timmerman, M. Crego-Calama, K. Versluis, A. J. R. Heck, I. Luyten, D. N. Reinhoudt *J. Am. Chem. Soc.* **2002**, *124*, 6569-6575.

5. NMR Spectra of All Complexes and Organic Products

4

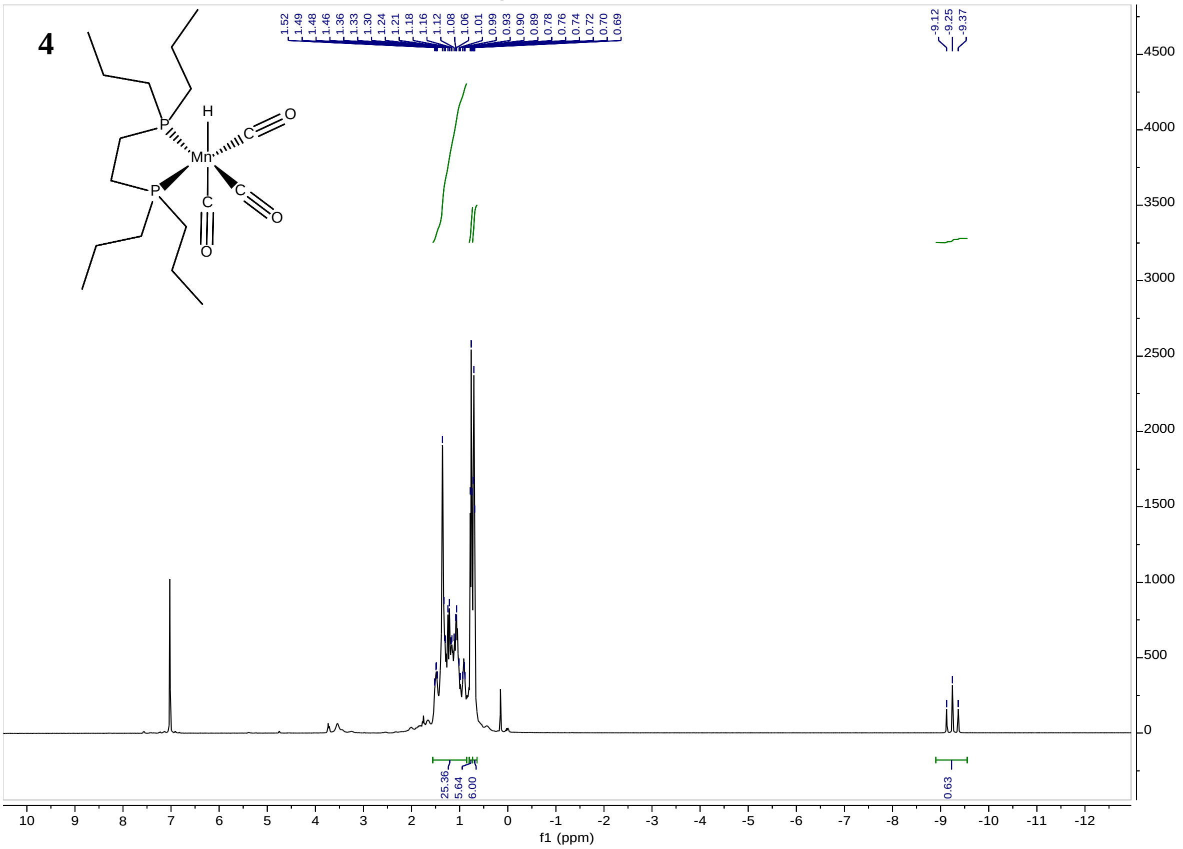

4

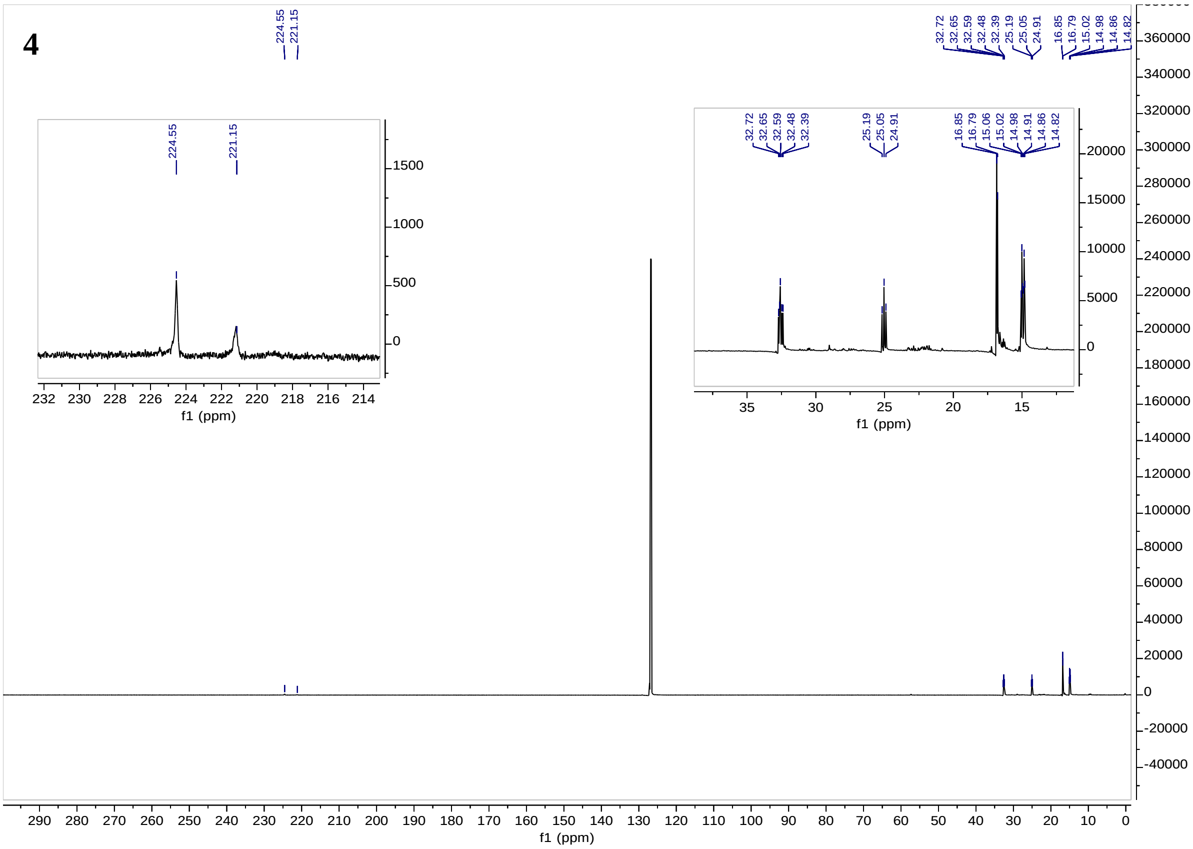

4

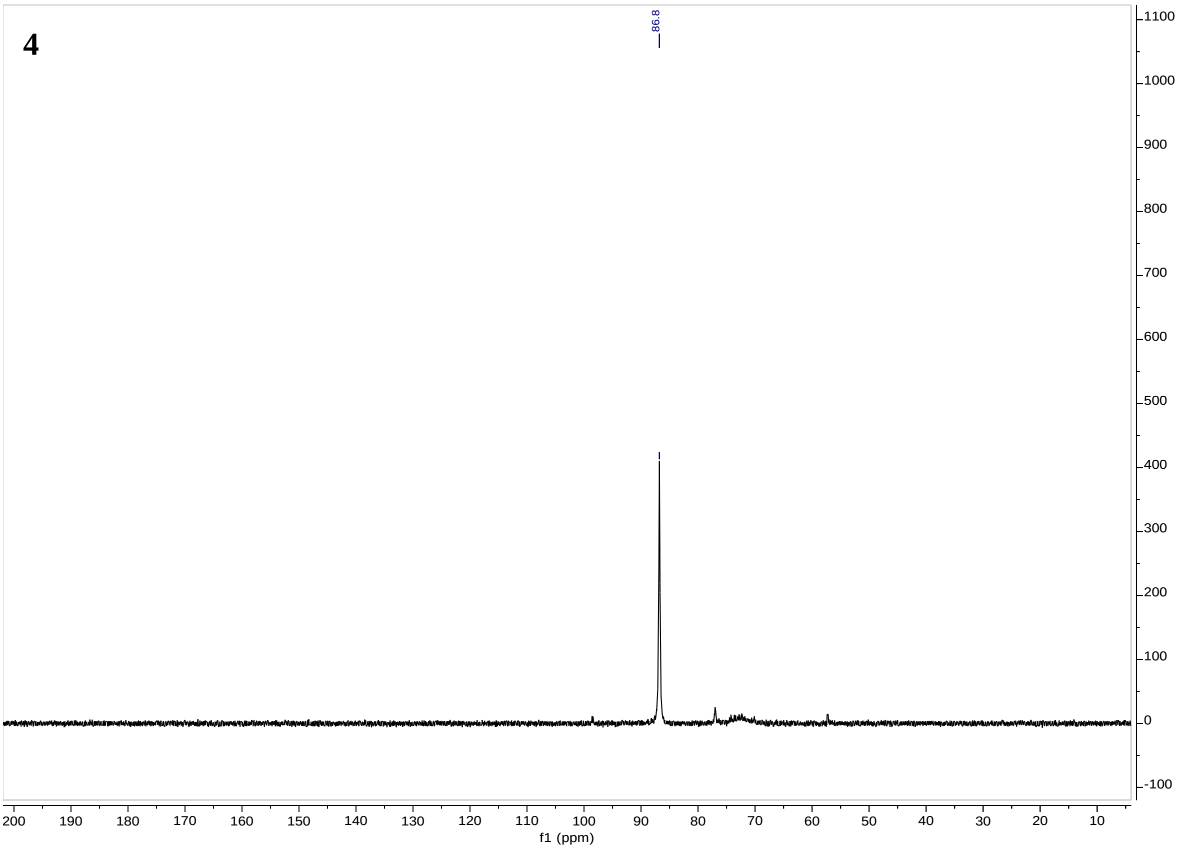
86.8
1100
1000
900
800
700
600
500
400
300
200
100
0
-100
200
190
180
170
160
150
140
130
120
110
100
90
80
70
60
50
40
30
20
10
f1 (ppm)

4

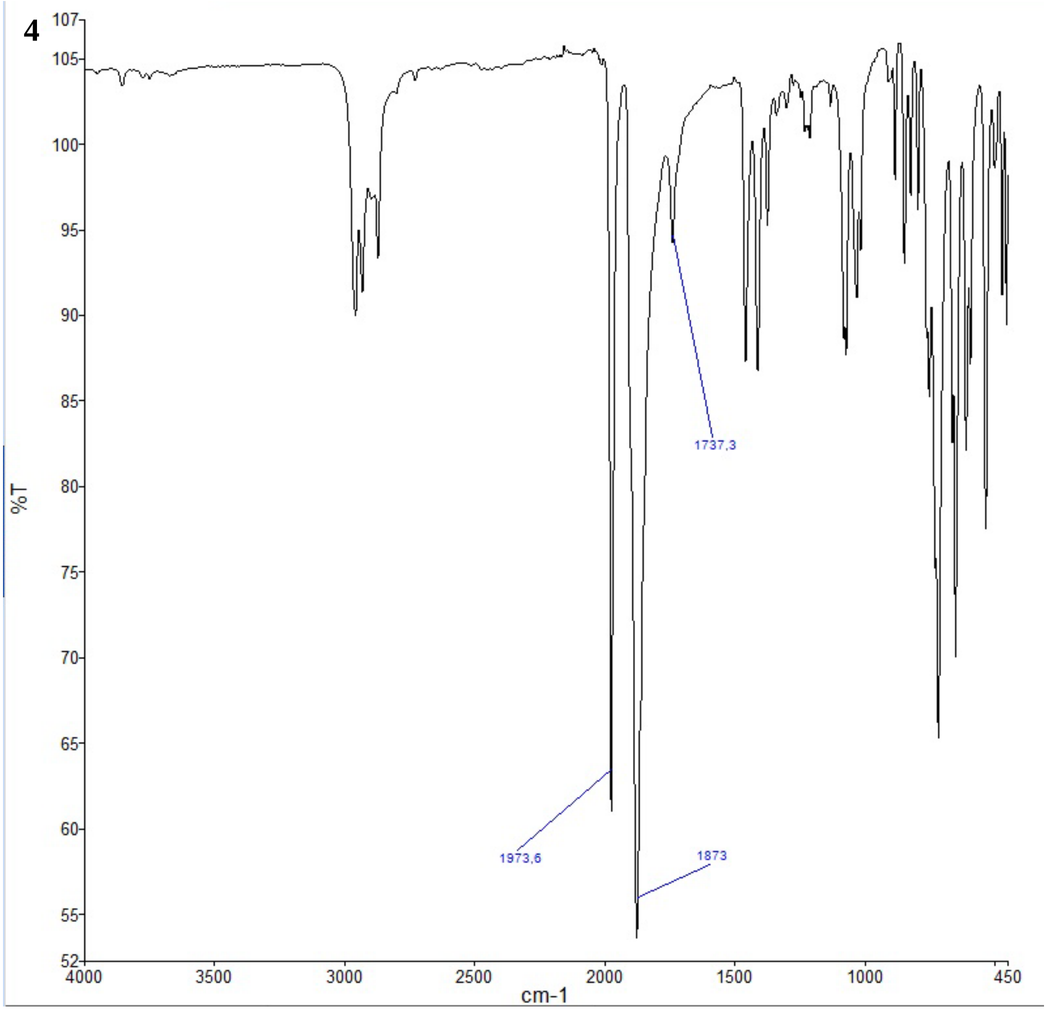
107
105
100
95
90
85
80
75
70
65
60
55
52
%T
1737,3
1973,6
1873
4000
3500
3000
2500
2000
1500
1000
450
cm-1

4

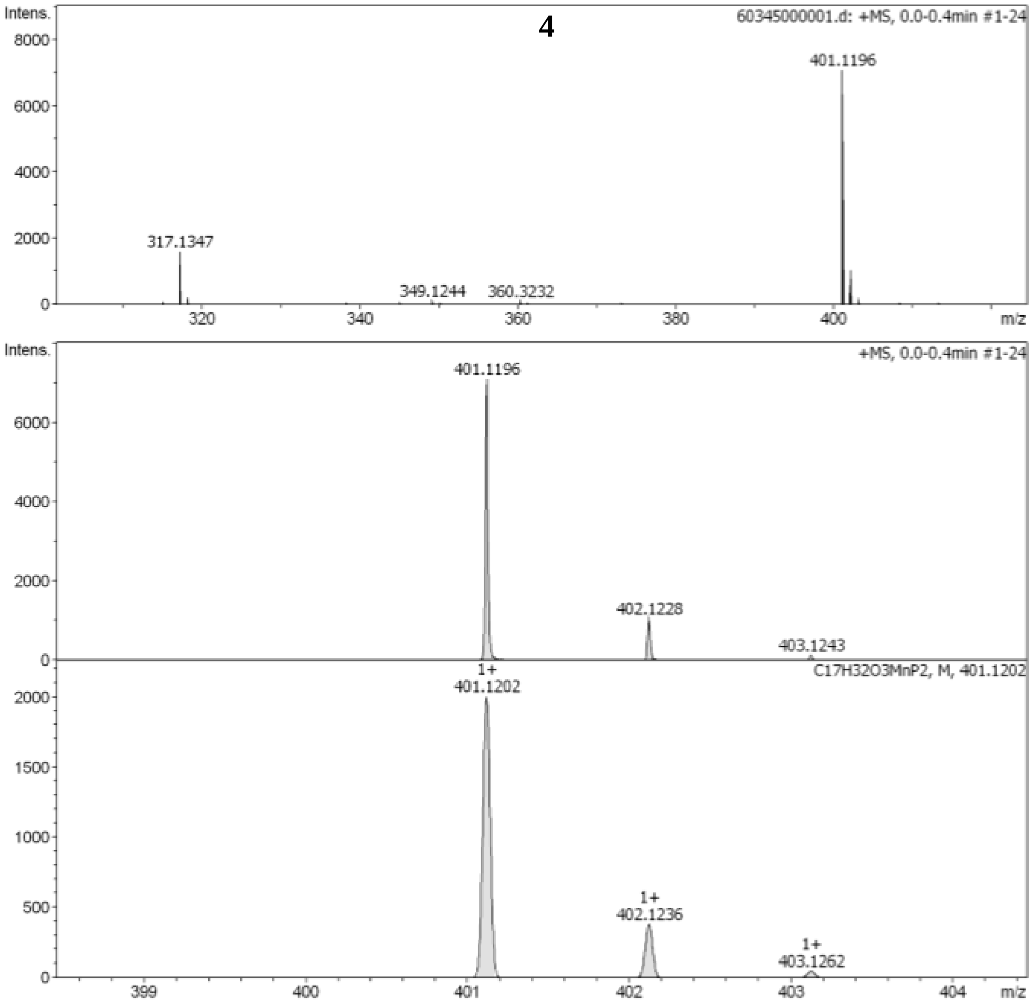

Intens.
60345000001.d: +MS, 0.0-0.4min #1-24
401.1196
317.1347
349.1244
360.3232
m/z
+MS, 0.0-0.4min #1-24
401.1196
402.1228
403.1243
C17H32O3MnP2, M, 401.1202
1+
401.1202
1+
402.1236
1+
403.1262
m/z

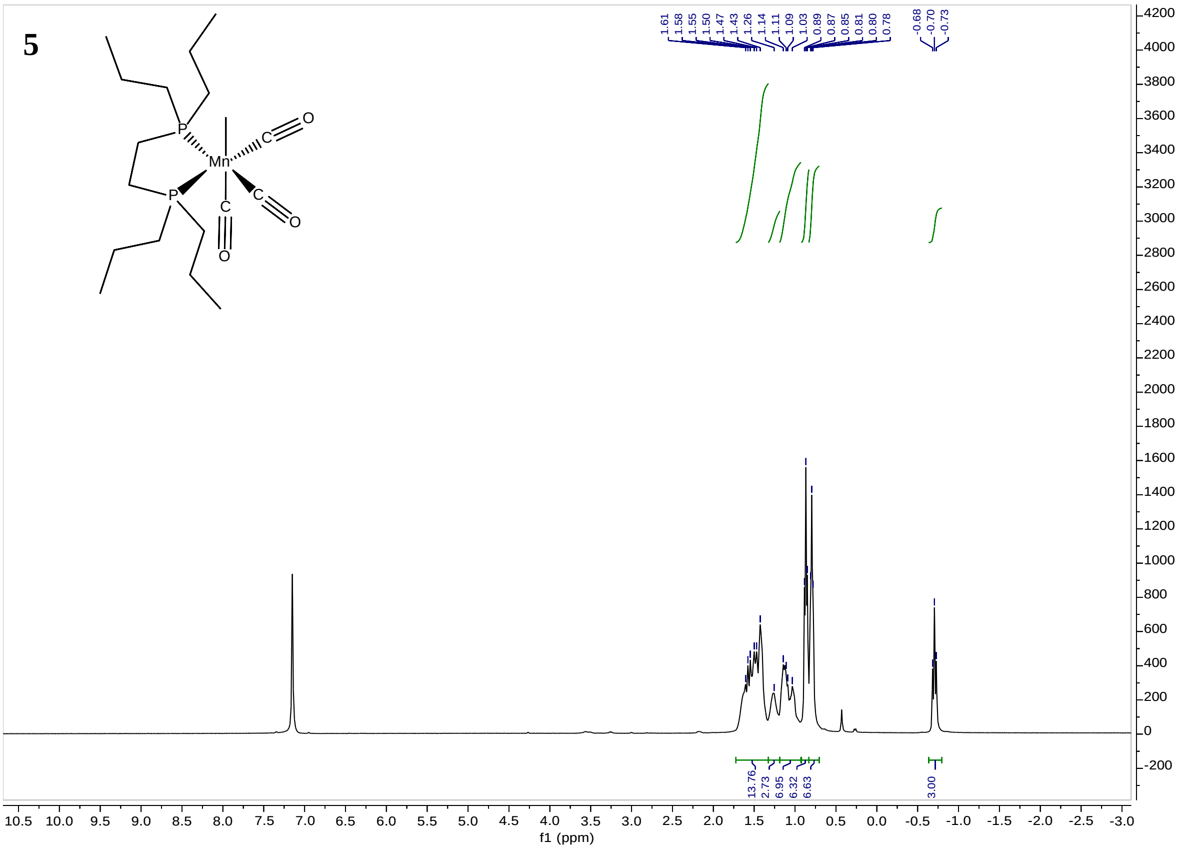
5
1.61
1.58
1.55
1.50
1.47
1.43
1.26
1.14
1.11
1.09
1.03
0.89
0.87
0.85
0.81
0.80
0.78
-0.68
-0.70
-0.73
13.76
2.73
6.95
6.32
6.63
3.00
f1 (ppm)

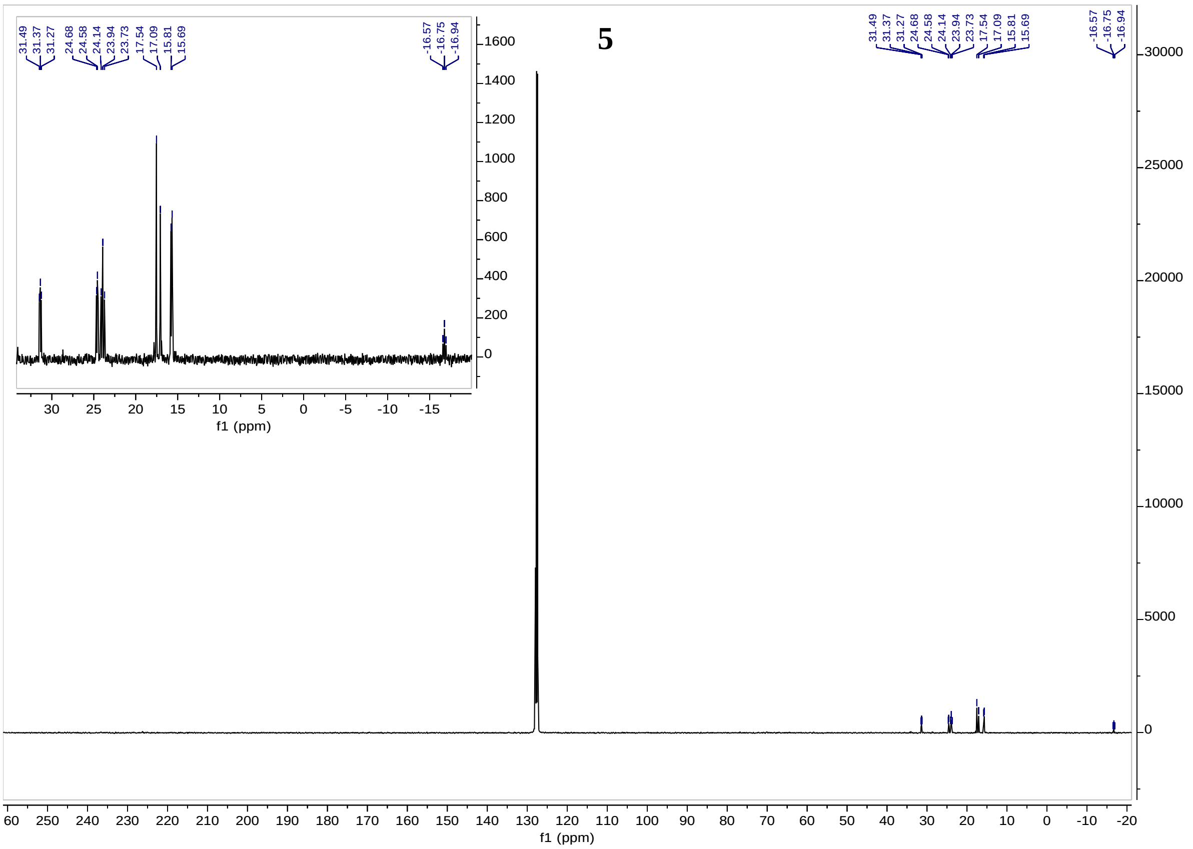
5
31.49
31.37
31.27
24.68
24.58
24.14
23.94
23.73
17.54
17.09
15.81
15.69
-16.57
-16.75
-16.94
f1 (ppm)

5

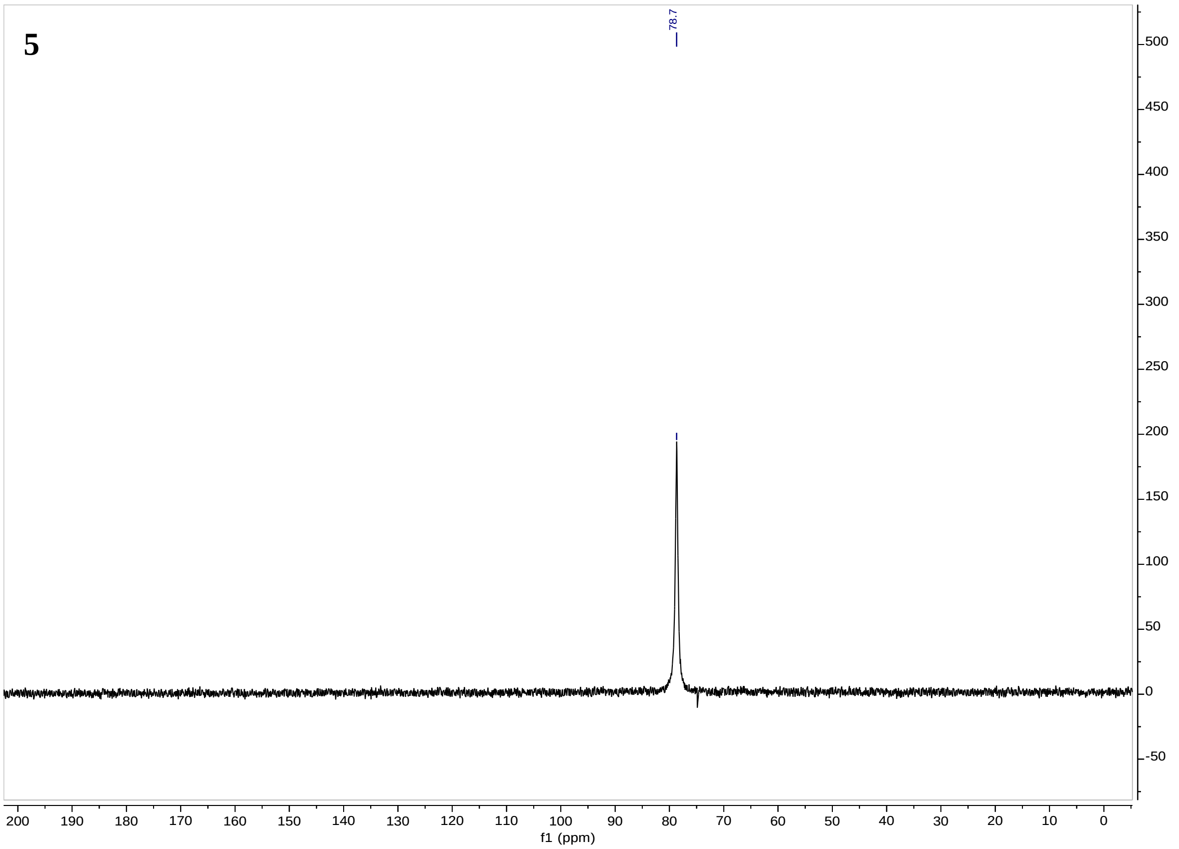

78.7
500
450
400
350
300
250
200
150
100
50
0
-50
200 190 180 170 160 150 140 130 120 110 100 90 80 70 60 50 40 30 20 10 0
f1 (ppm)

5

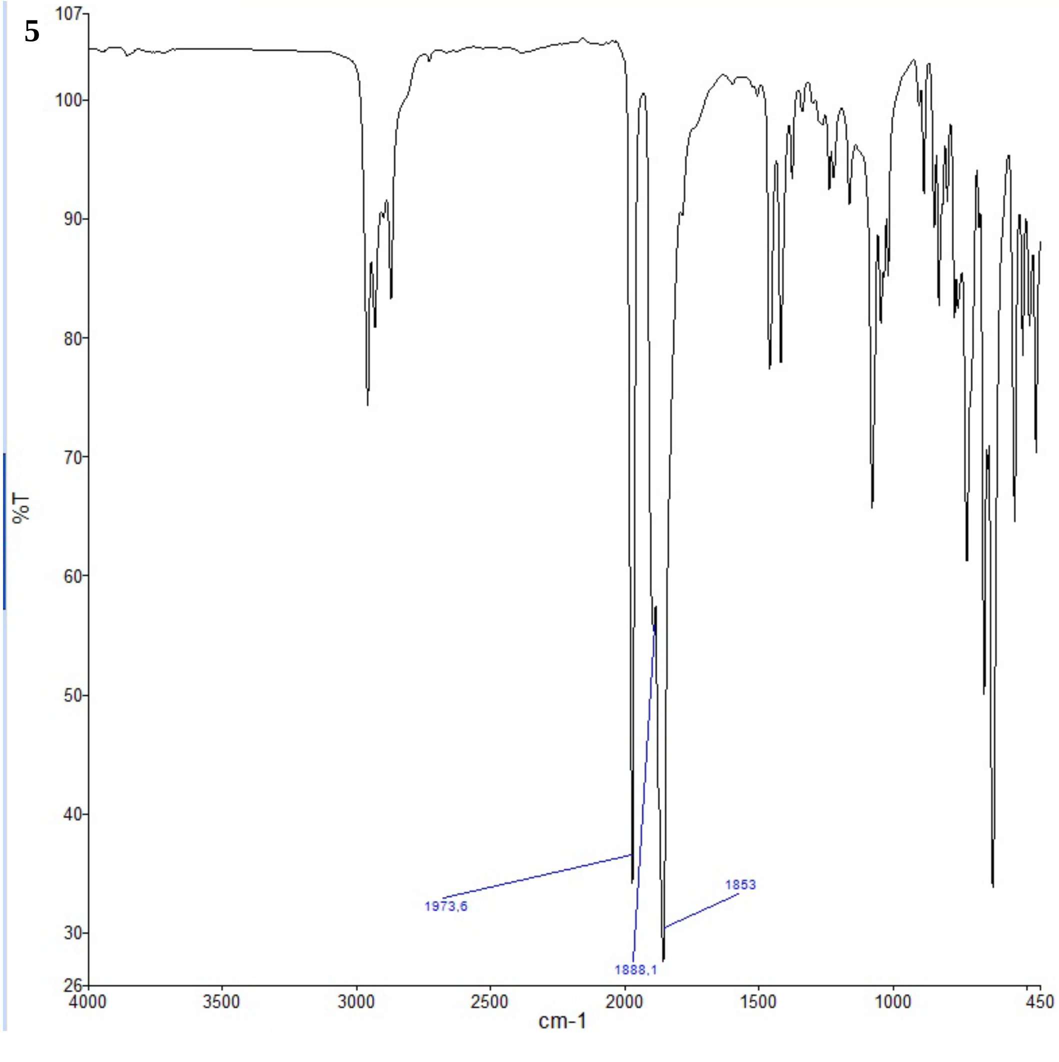

107
100
90
80
70
60
50
40
30
26
%T
1973,6
1888,1
1853
4000 3500 3000 2500 2000 1500 1000 450
cm-1

5

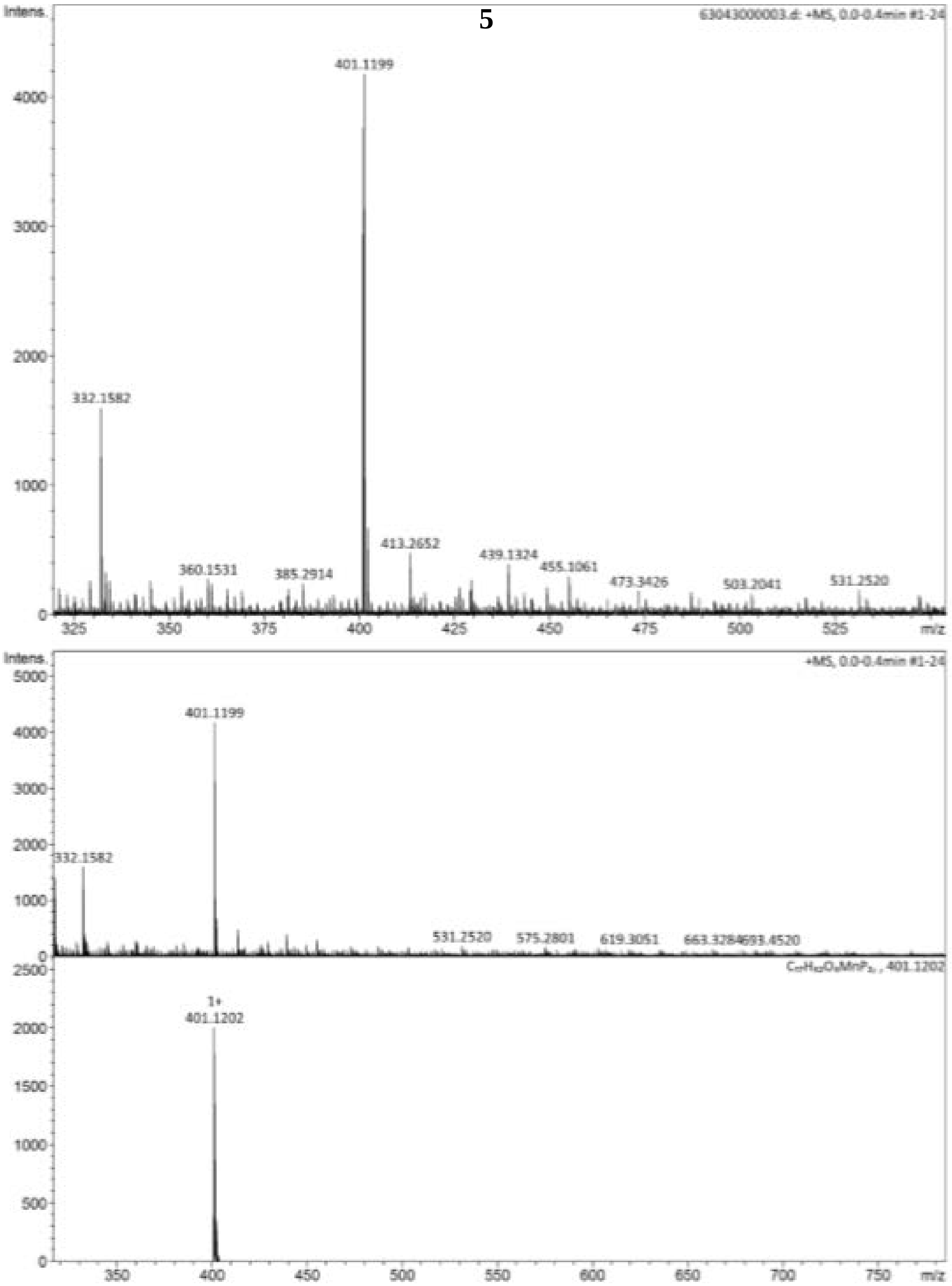
Intens.
63043000003.d: +MS, 0.0-0.4min #1-24
401.1199
332.1582
360.1531
385.2914
413.2652
439.1324
455.1061
473.3426
503.2041
531.2520
m/z
+MS, 0.0-0.4min #1-24
575.2801
619.3051
663.3284
693.4520
1+
401.1202

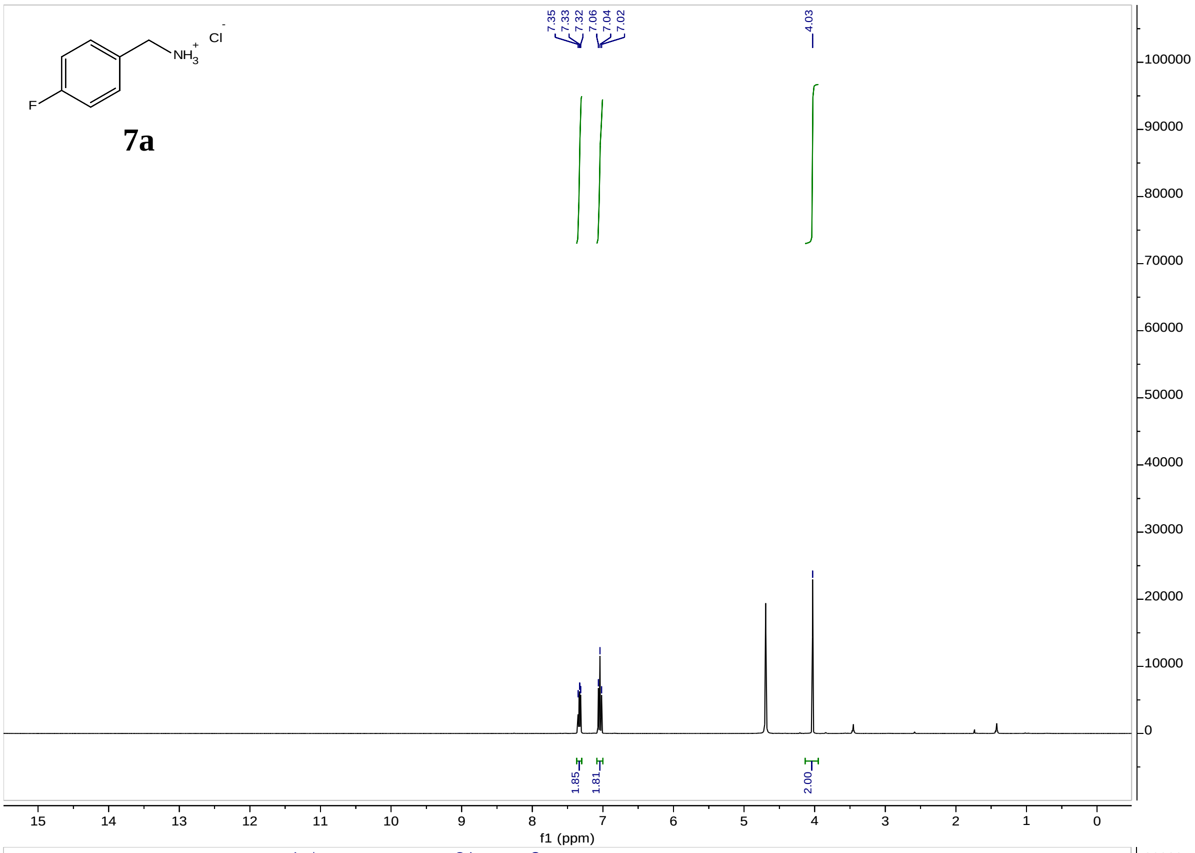
F
NH3+
Cl-
7a
7.35
7.33
7.32
7.06
7.04
7.02
4.03
1.85
1.81
2.00
f1 (ppm)

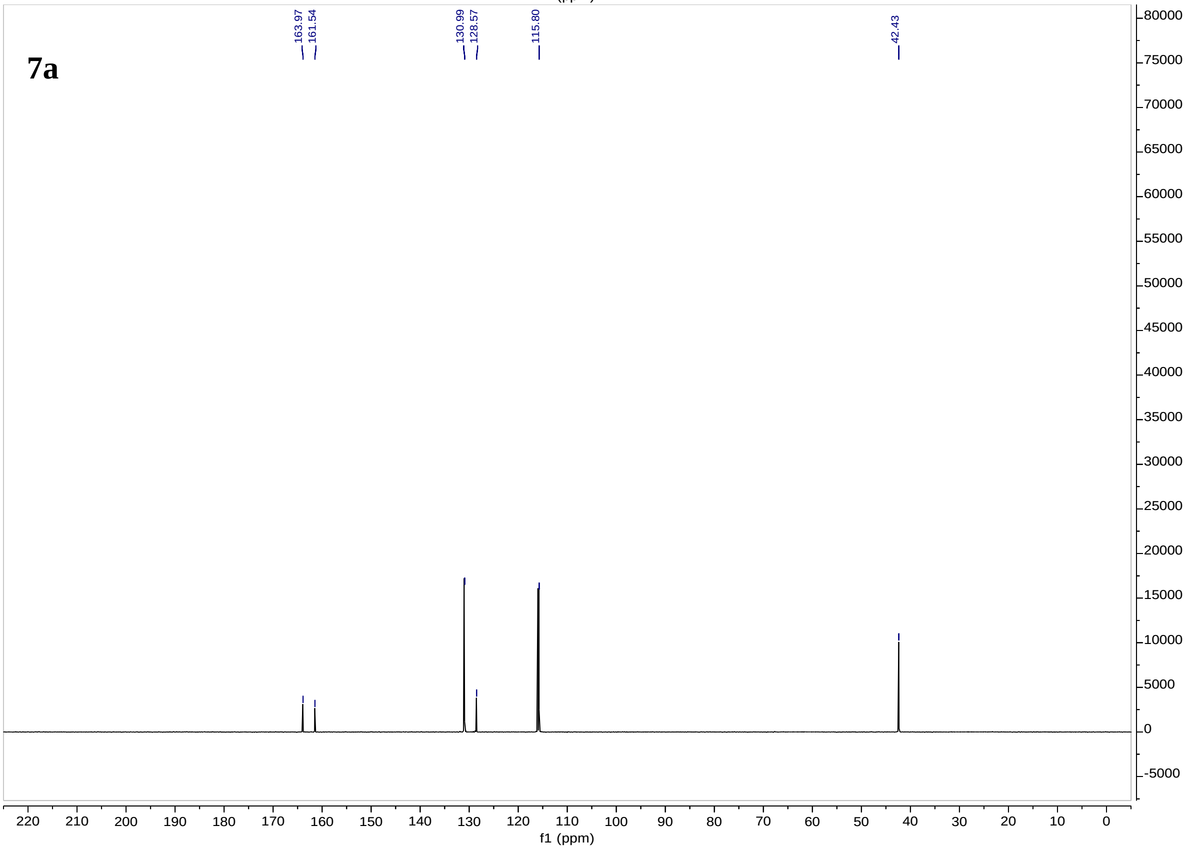
7a
163.97
161.54
130.99
128.57
115.80
42.43
f1 (ppm)

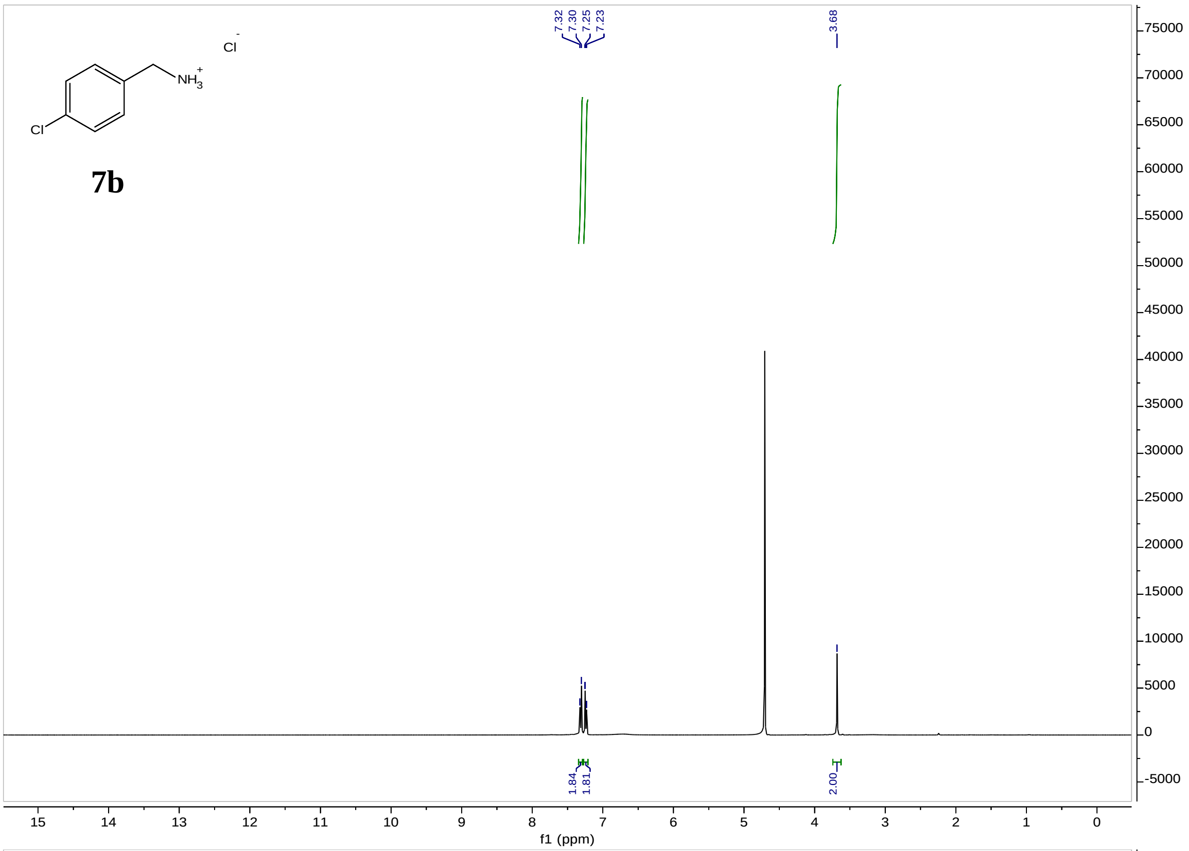
Cl
NH3+
Cl-
7.32
7.30
7.25
7.23
3.68
1.84
1.81
2.00
f1 (ppm)

7b

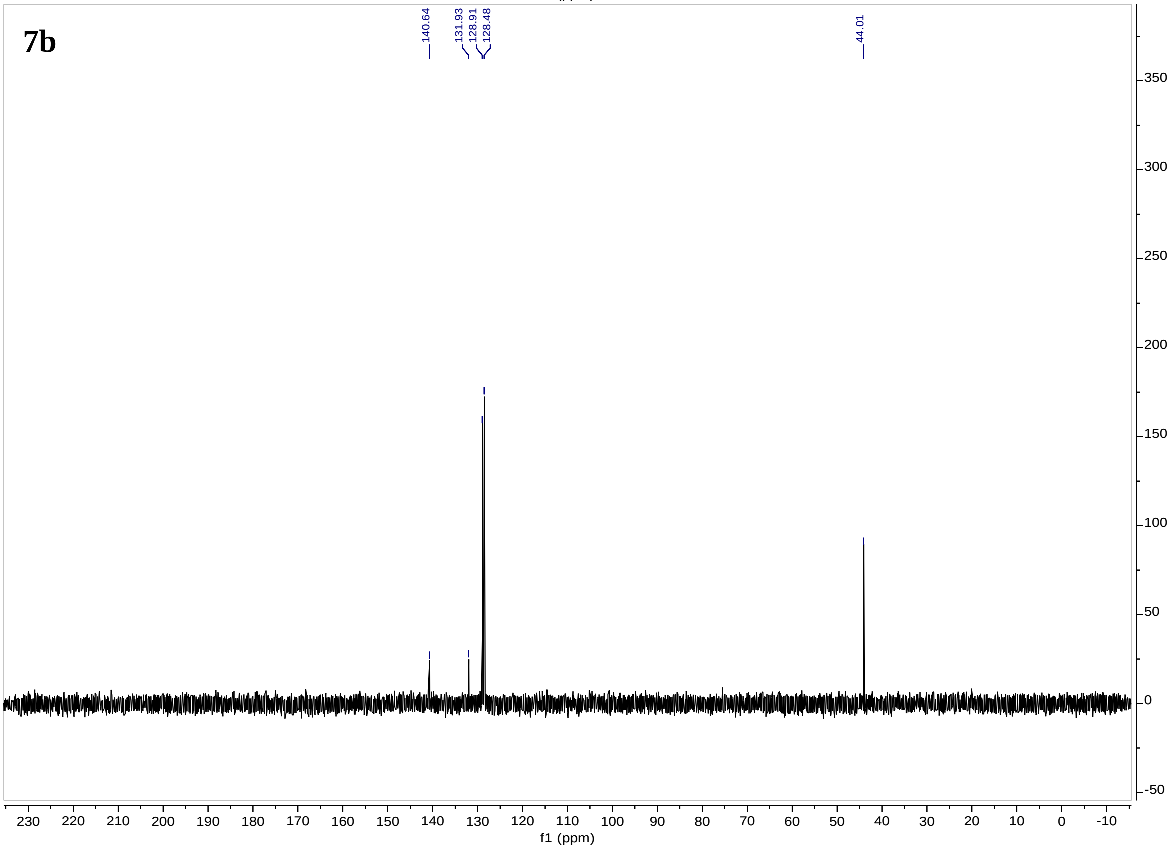
140.64
131.93
128.91
128.48
44.01
f1 (ppm)

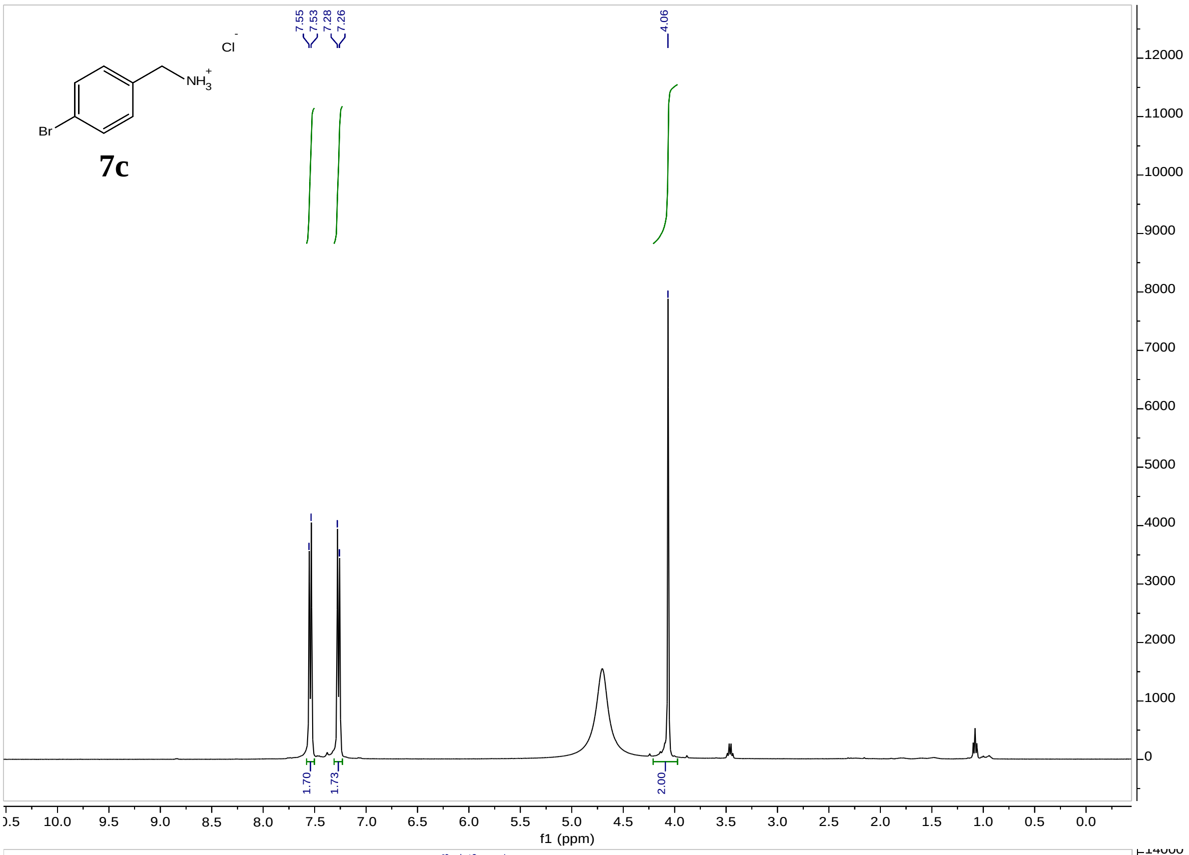

7c

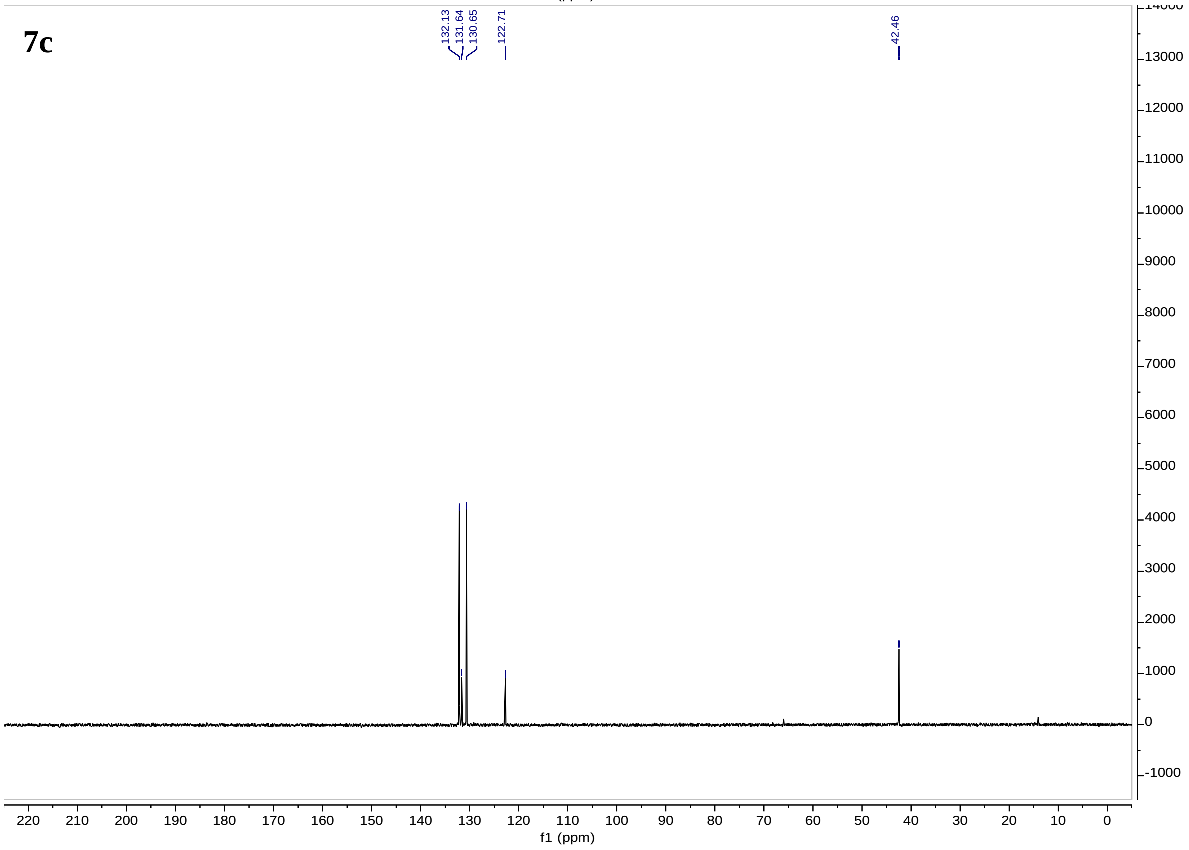

7c

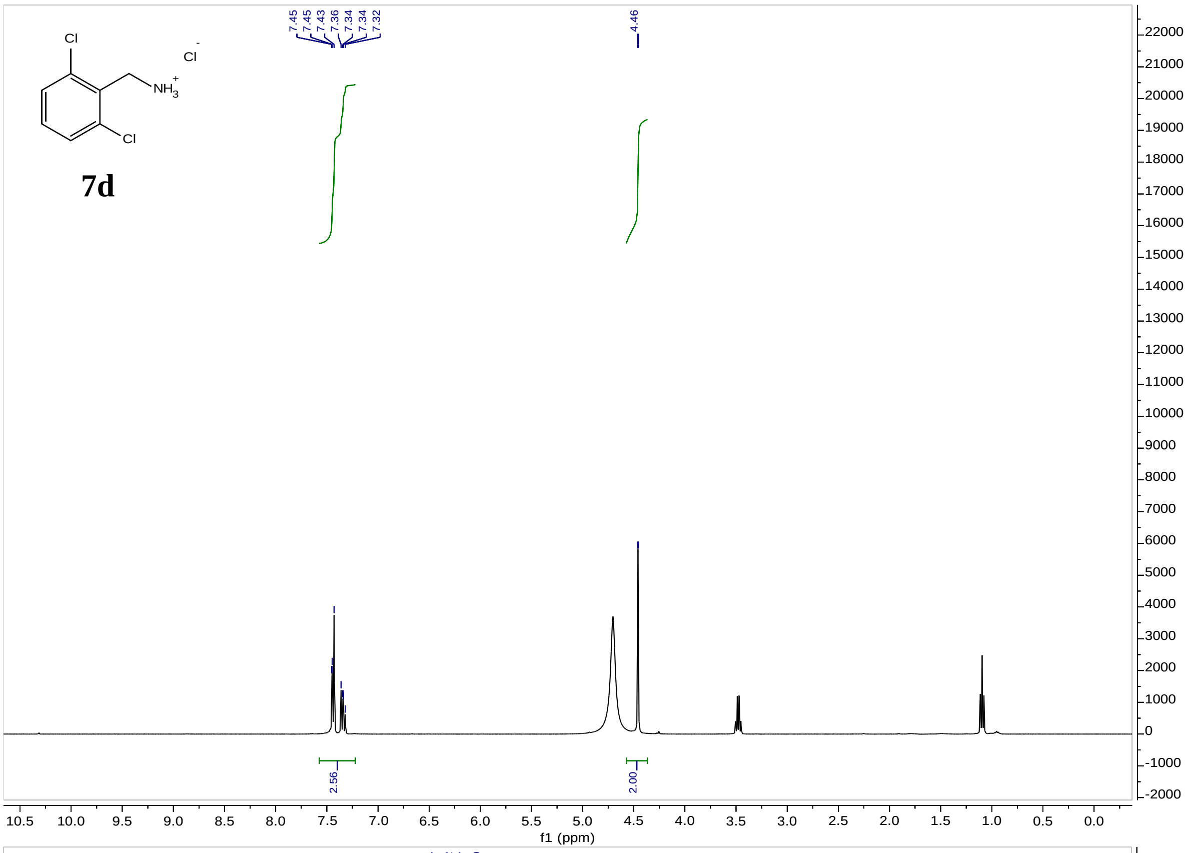
7d
7.45
7.45
7.43
7.36
7.34
7.34
7.32
4.46
2.56
2.00
f1 (ppm)

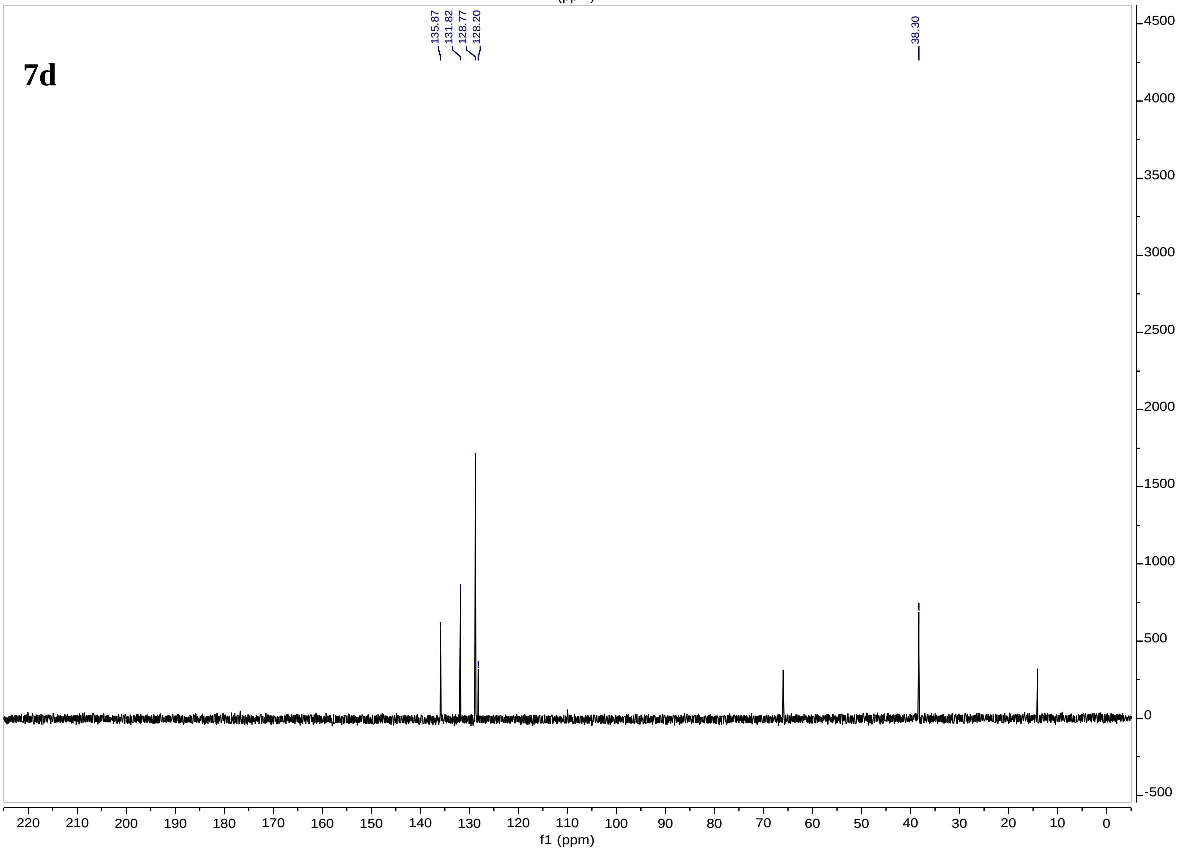
7d
135.87
131.82
128.77
128.20
38.30
f1 (ppm)

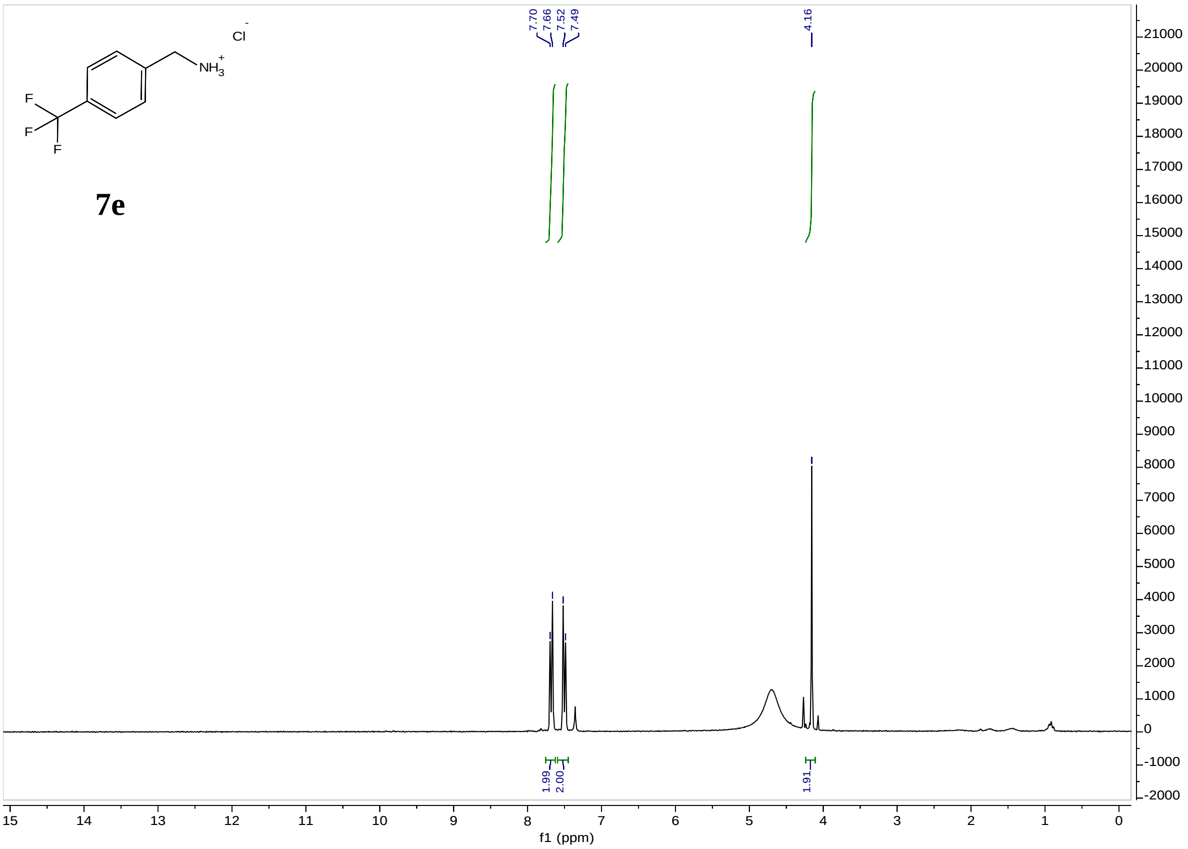
Cl-
NH3+
F
F
F
7.70
7.66
7.52
7.49
4.16
1.99
2.00
1.91
f1 (ppm)

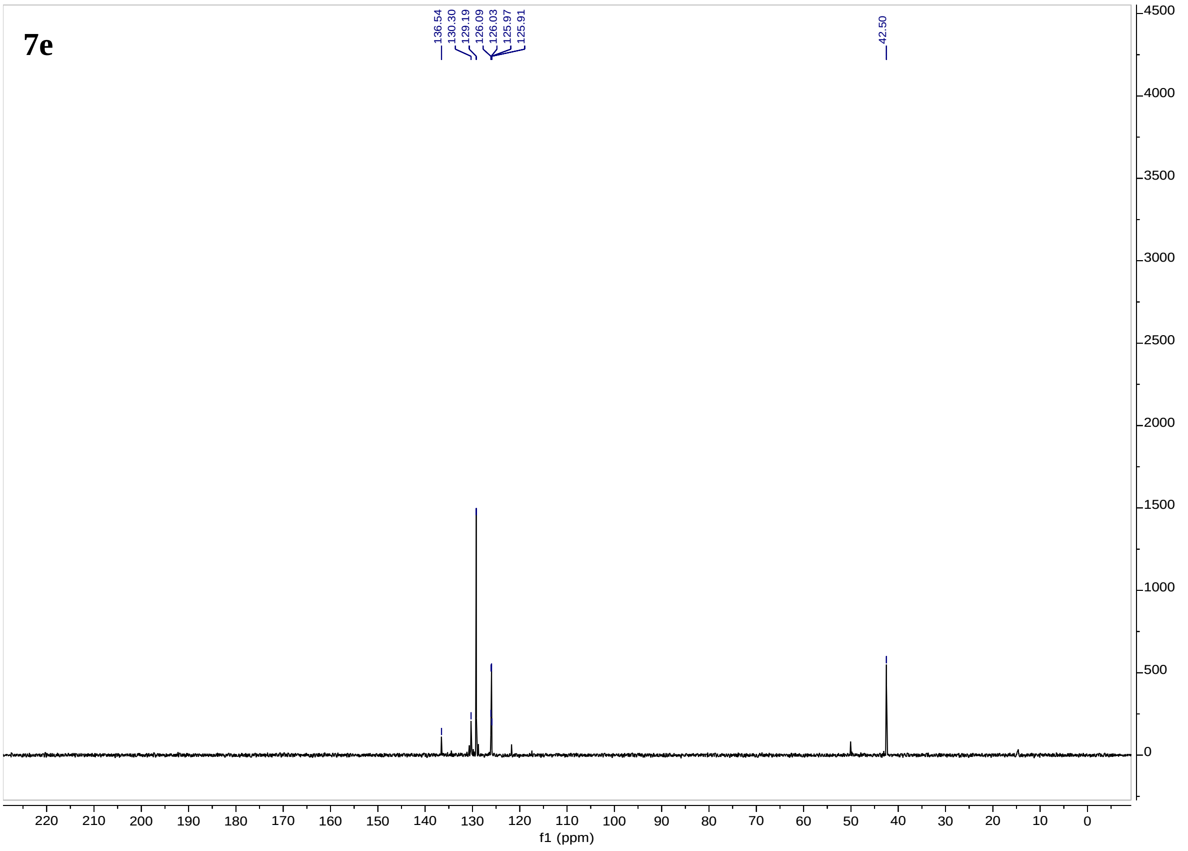
7e
136.54
130.30
129.19
126.09
126.03
125.97
125.91
42.50
f1 (ppm)

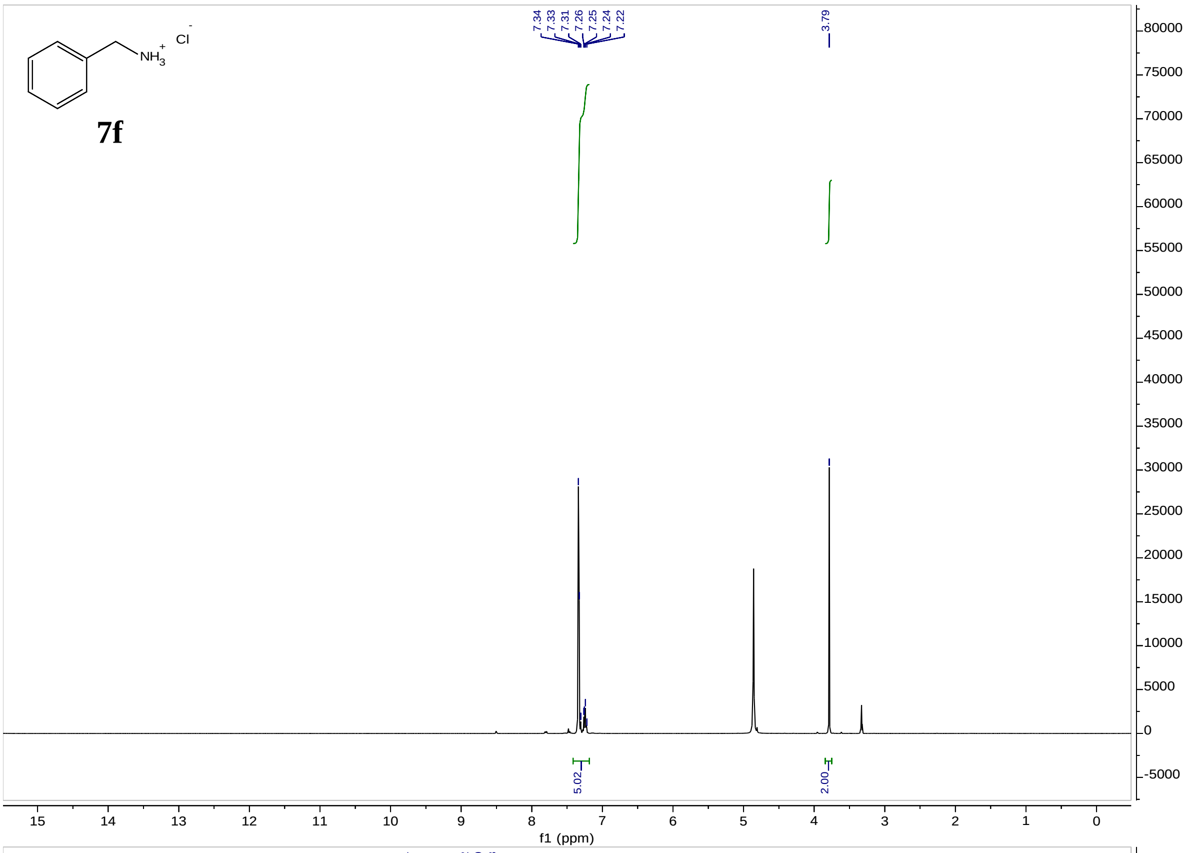
7f
NH3+ Cl-
7.34
7.33
7.31
7.26
7.25
7.24
7.22
3.79
5.02
2.00
f1 (ppm)

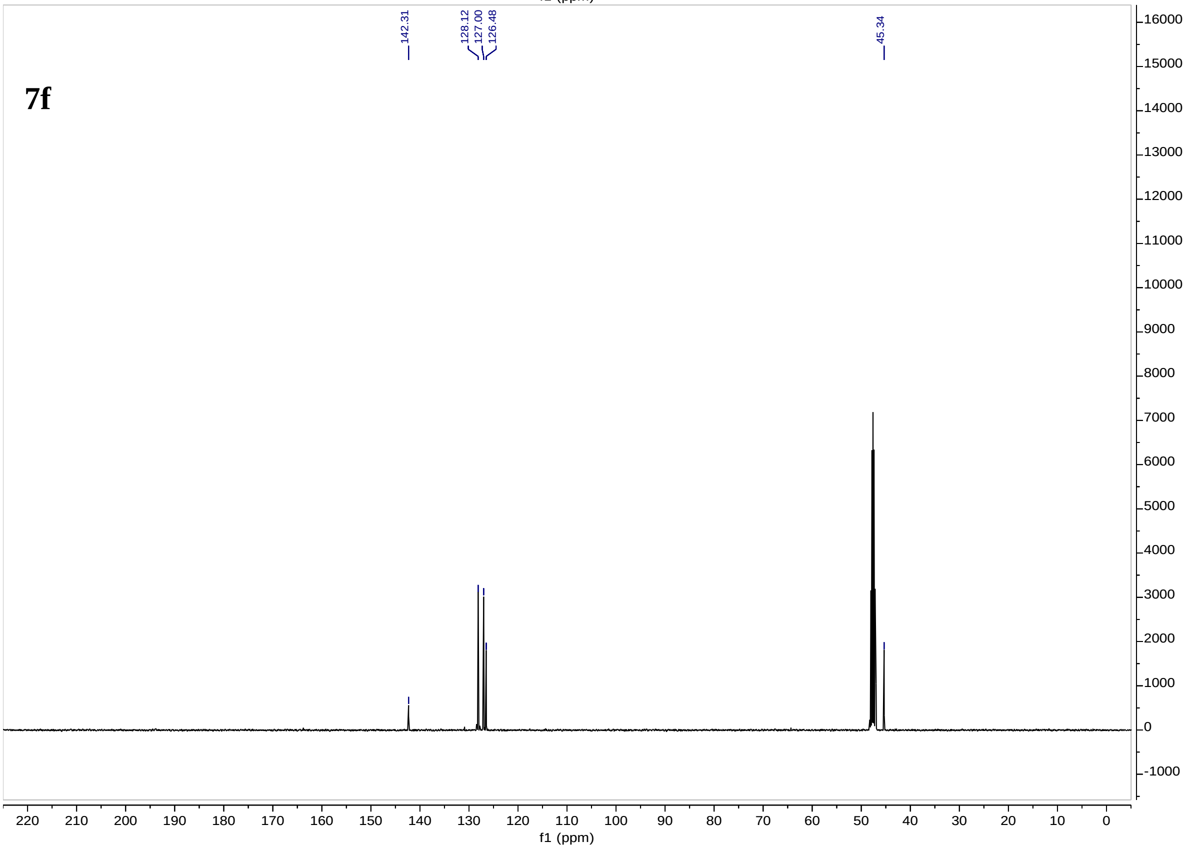
7f
142.31
128.12
127.00
126.48
45.34
f1 (ppm)

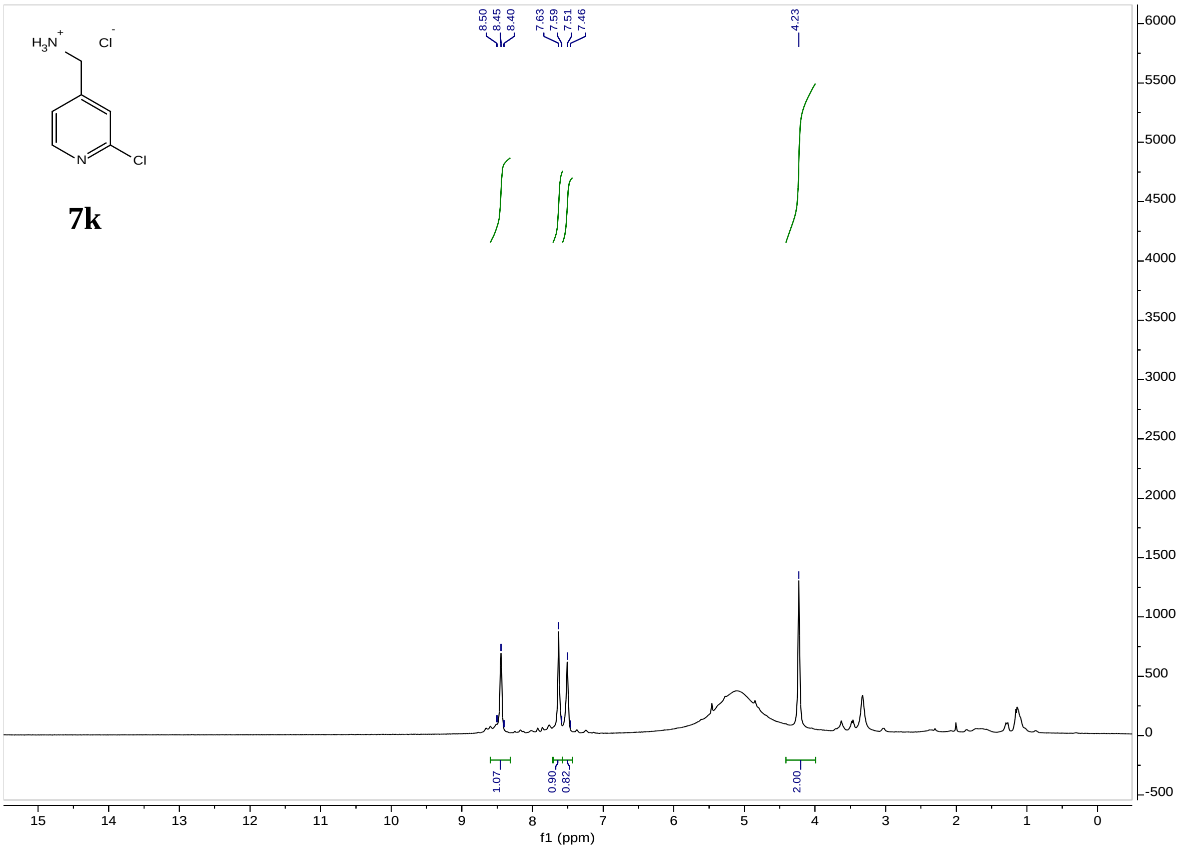

H3N+ Cl-
N Cl
7k
8.50
8.45
8.40
7.63
7.59
7.51
7.46
4.23
1.07
0.90
0.82
2.00
f1 (ppm)

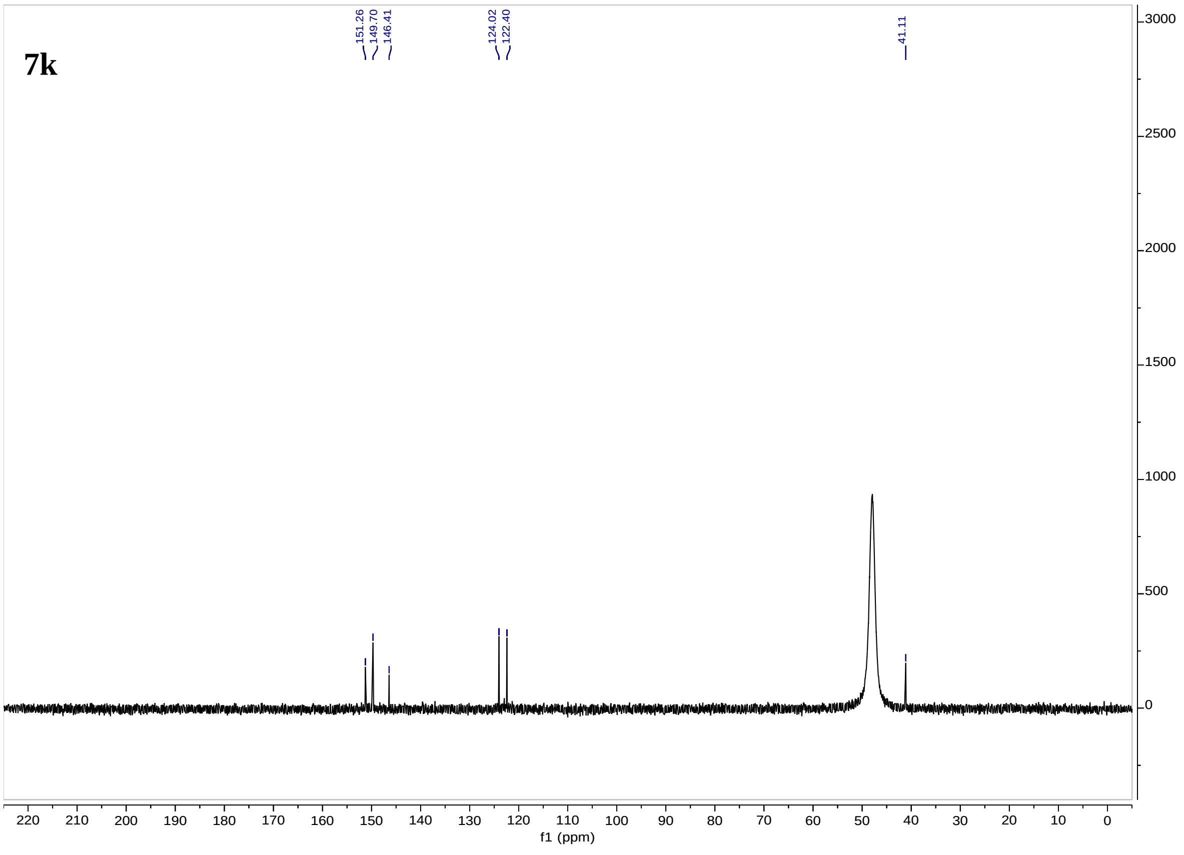

7k
151.26
149.70
146.41
124.02
122.40
41.11
f1 (ppm)

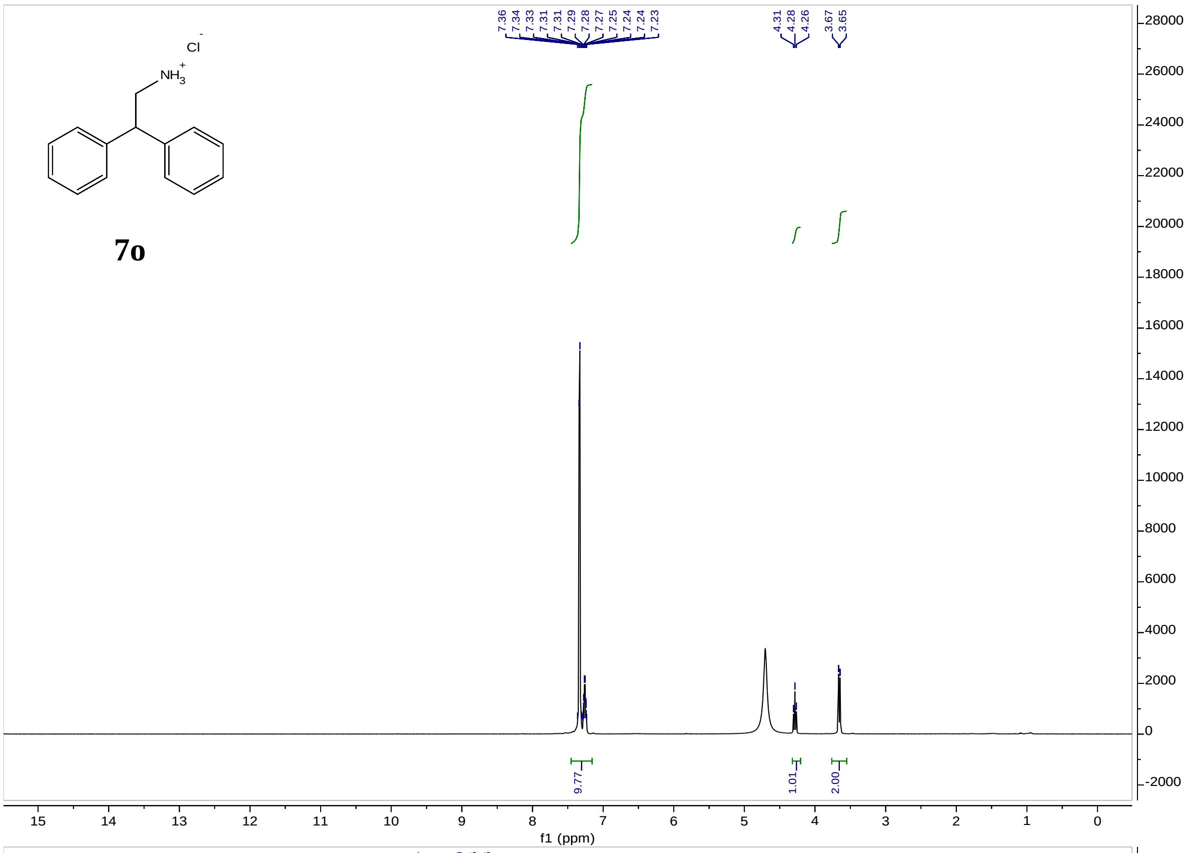
Cl-
NH3+
7o
7.36
7.34
7.33
7.31
7.31
7.29
7.28
7.27
7.25
7.24
7.24
7.23
4.31
4.28
4.26
3.67
3.65
9.77
1.01
2.00
f1 (ppm)

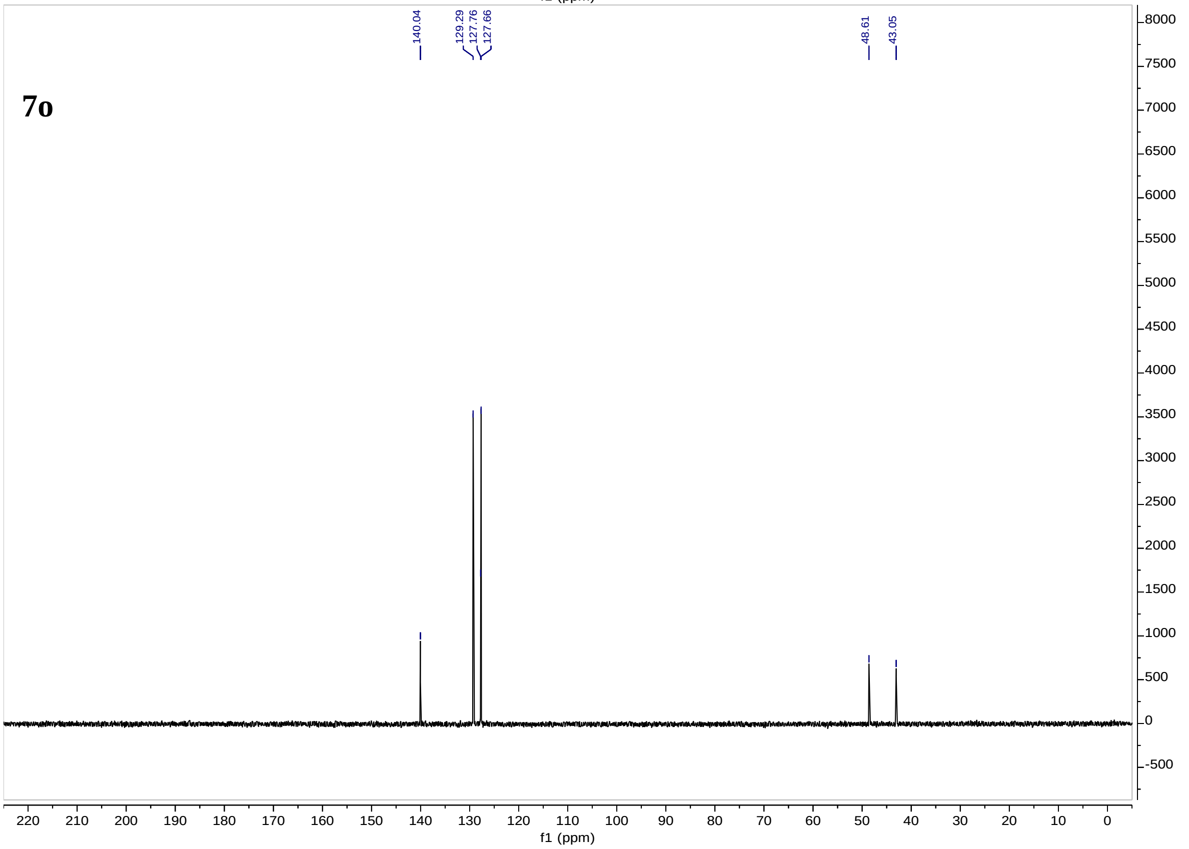
7o
140.04
129.29
127.76
127.66
48.61
43.05
f1 (ppm)

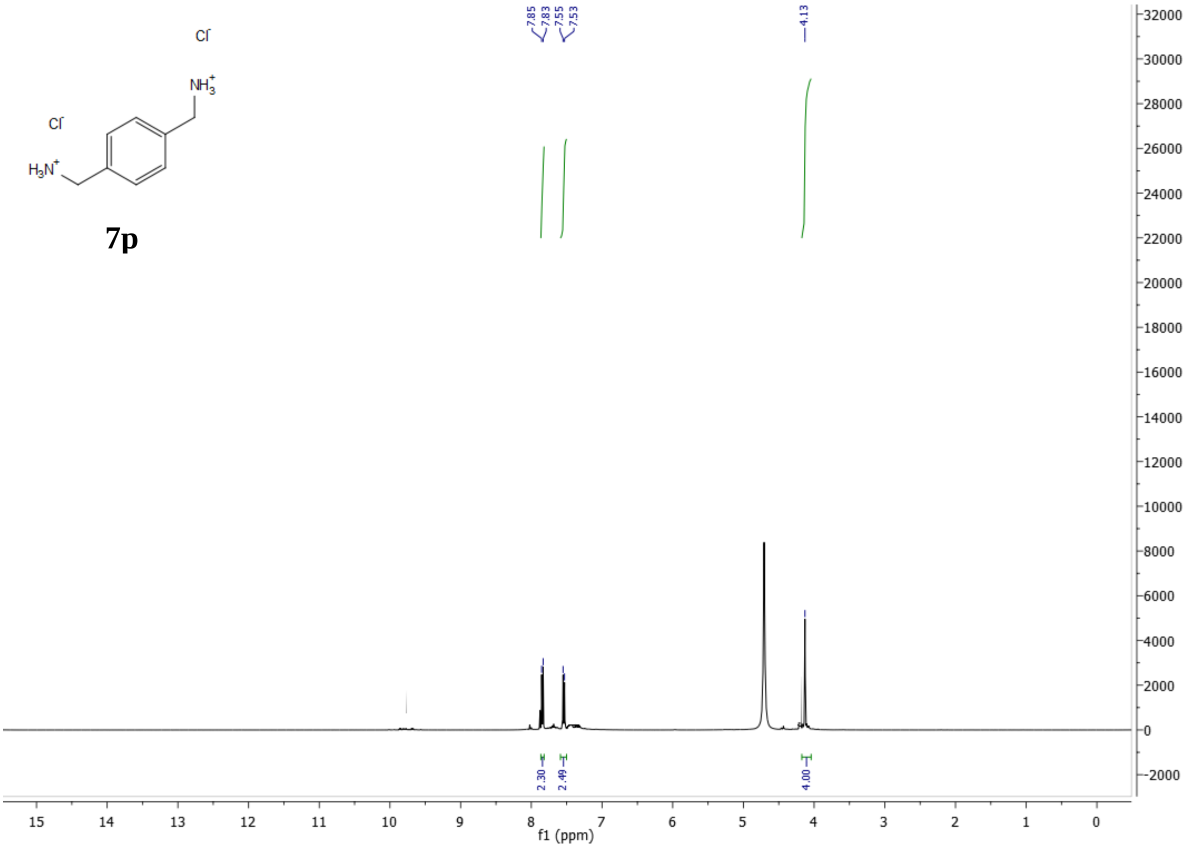
7p
7.85
7.83
7.55
7.53
4.13
2.30
2.49
4.00
f1 (ppm)

7p

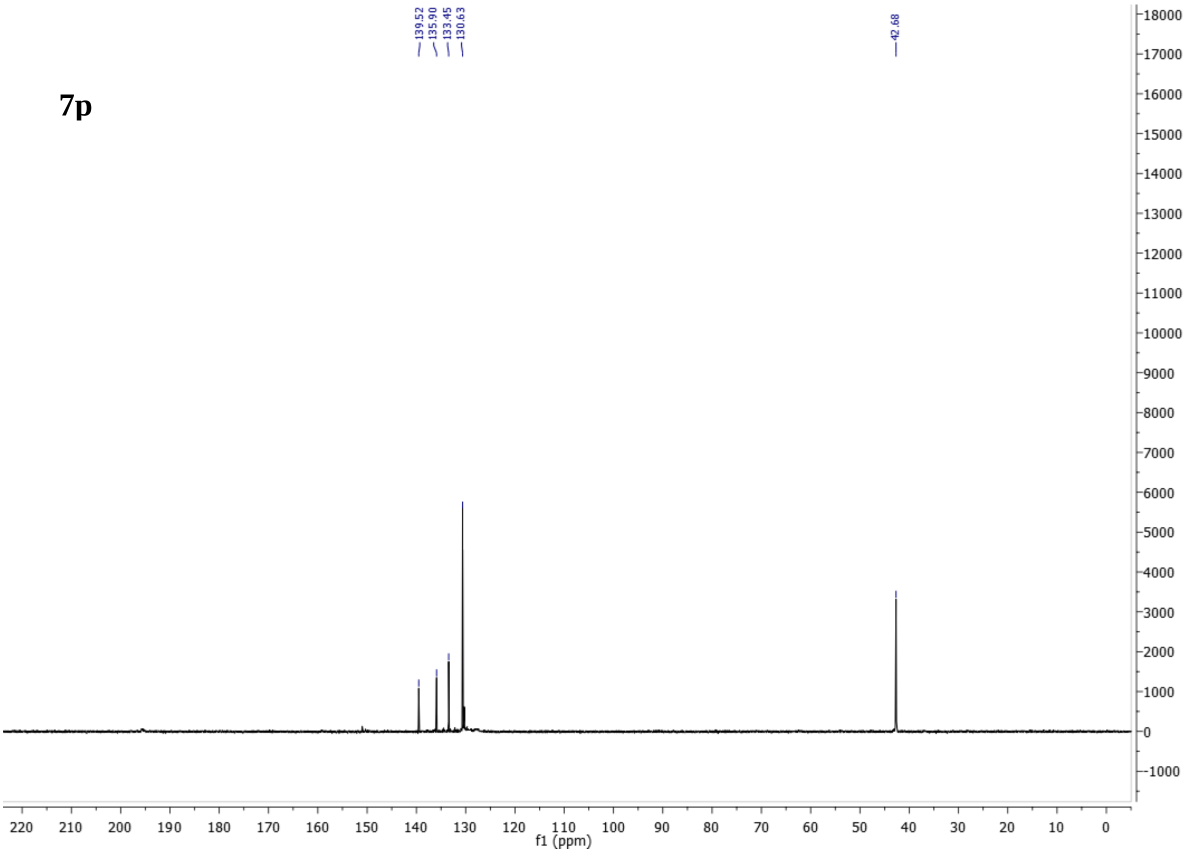
139.52
135.90
133.45
130.63
42.68
f1 (ppm)

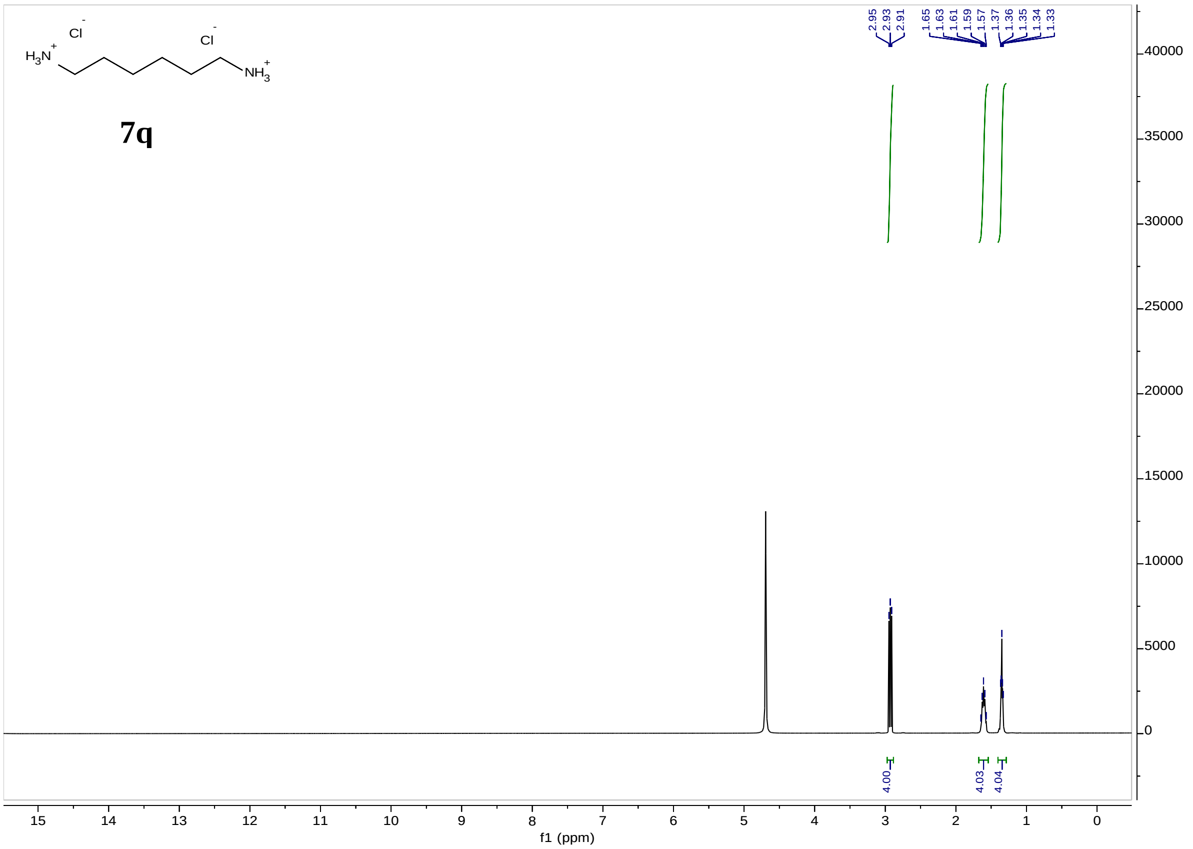
H3N+
Cl-
Cl-
NH3+
7q
2.95
2.93
2.91
1.65
1.63
1.61
1.59
1.57
1.37
1.36
1.35
1.34
1.33
4.00
4.03
4.04
f1 (ppm)

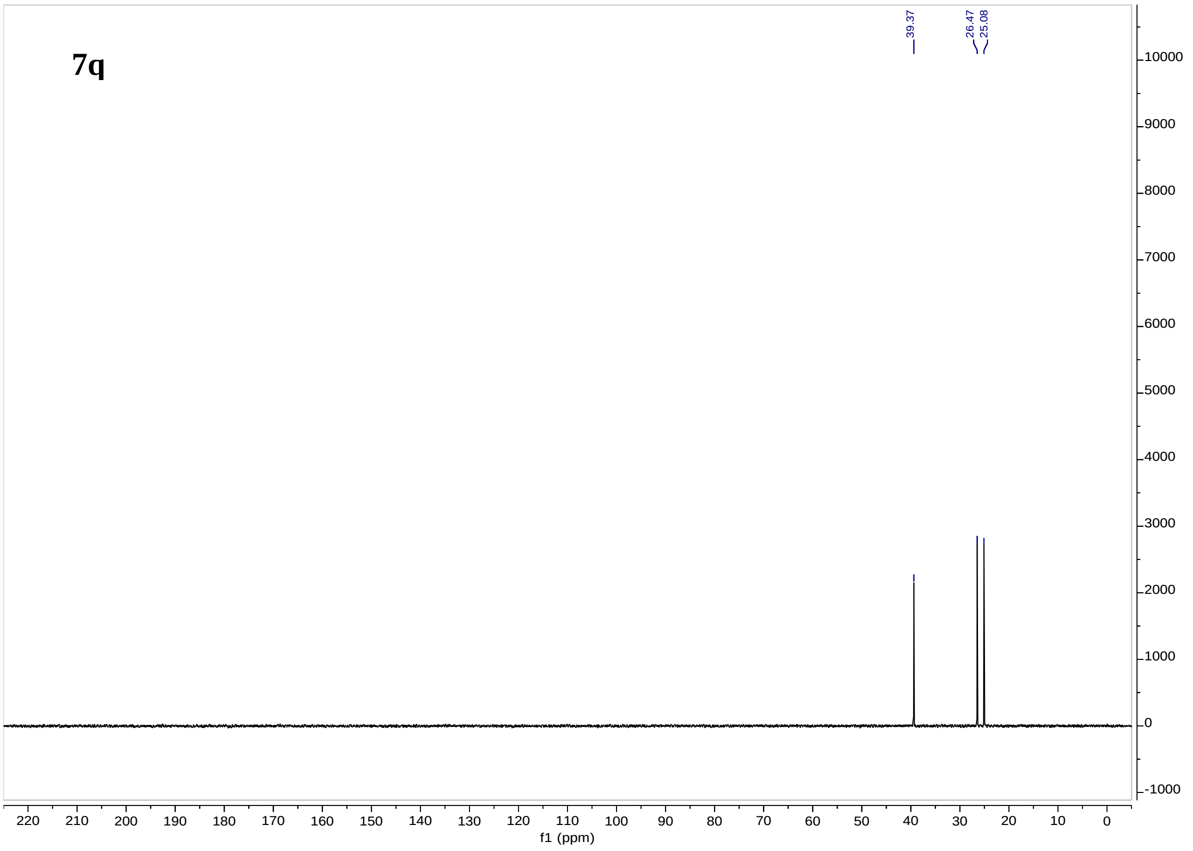
7q
39.37
26.47
25.08
f1 (ppm)

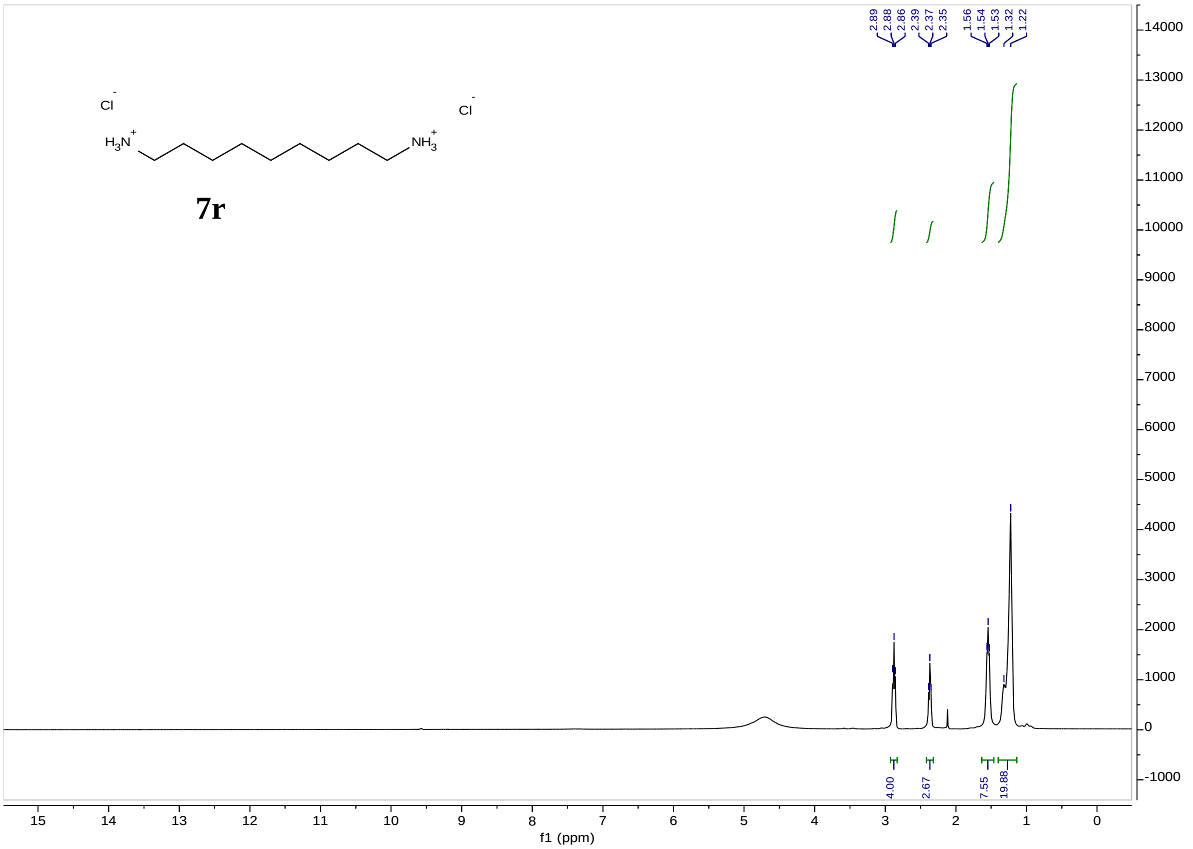
7r
2.89
2.88
2.86
2.39
2.37
2.35
1.56
1.54
1.53
1.32
1.22
4.00
2.67
7.55
19.88
f1 (ppm)

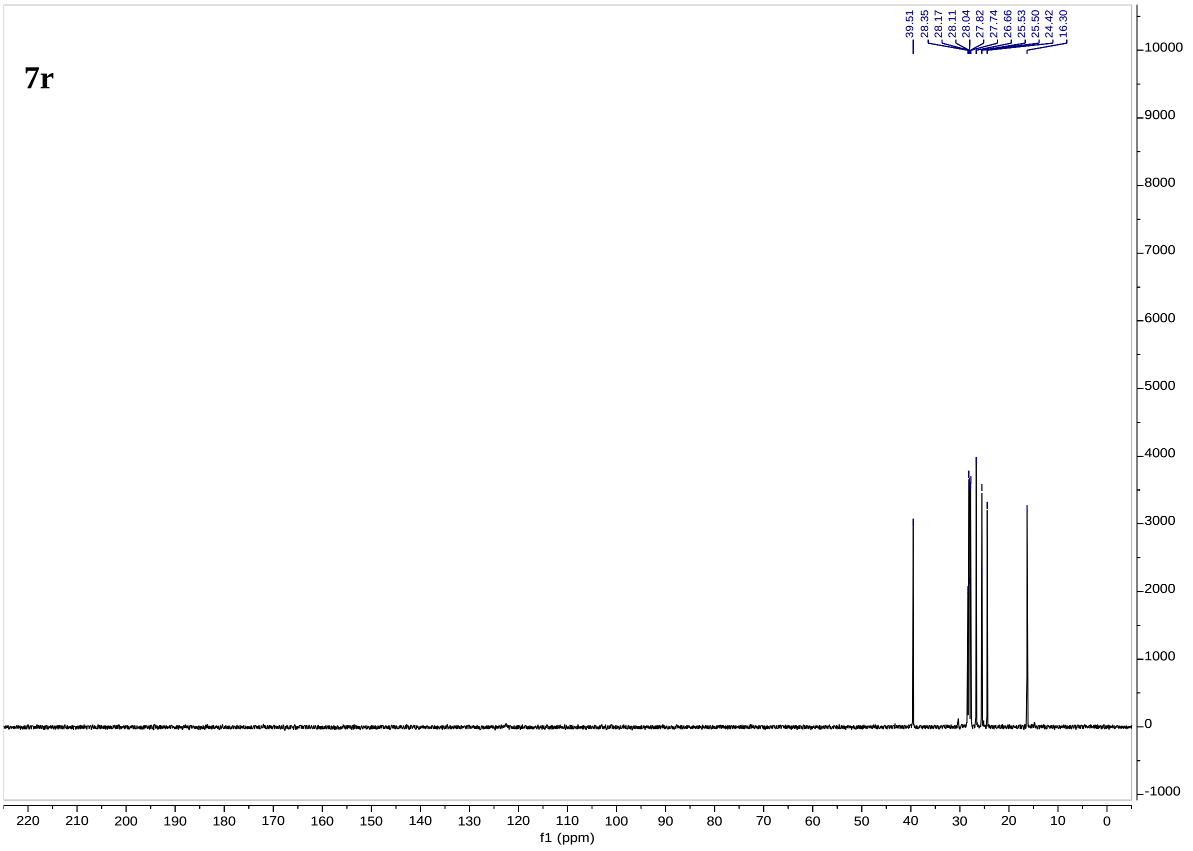
7r
39.51
28.35
28.17
28.11
28.04
27.82
27.74
26.66
25.53
25.50
24.42
16.30
f1 (ppm)

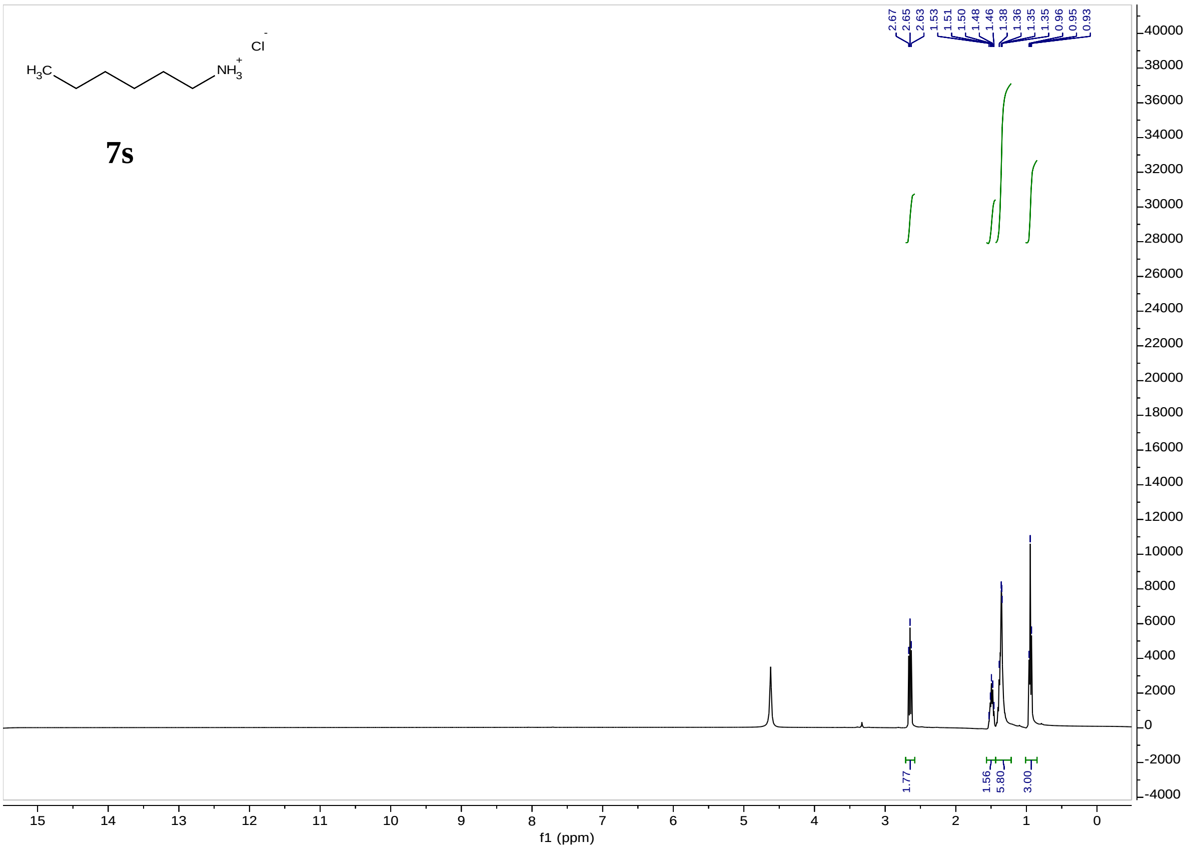

2.67
2.65
2.63
1.53
1.51
1.50
1.48
1.46
1.38
1.36
1.35
1.35
0.96
0.95
0.93
H3C
NH3+
Cl-
1.77
1.56
5.80
3.00
f1 (ppm)

7s

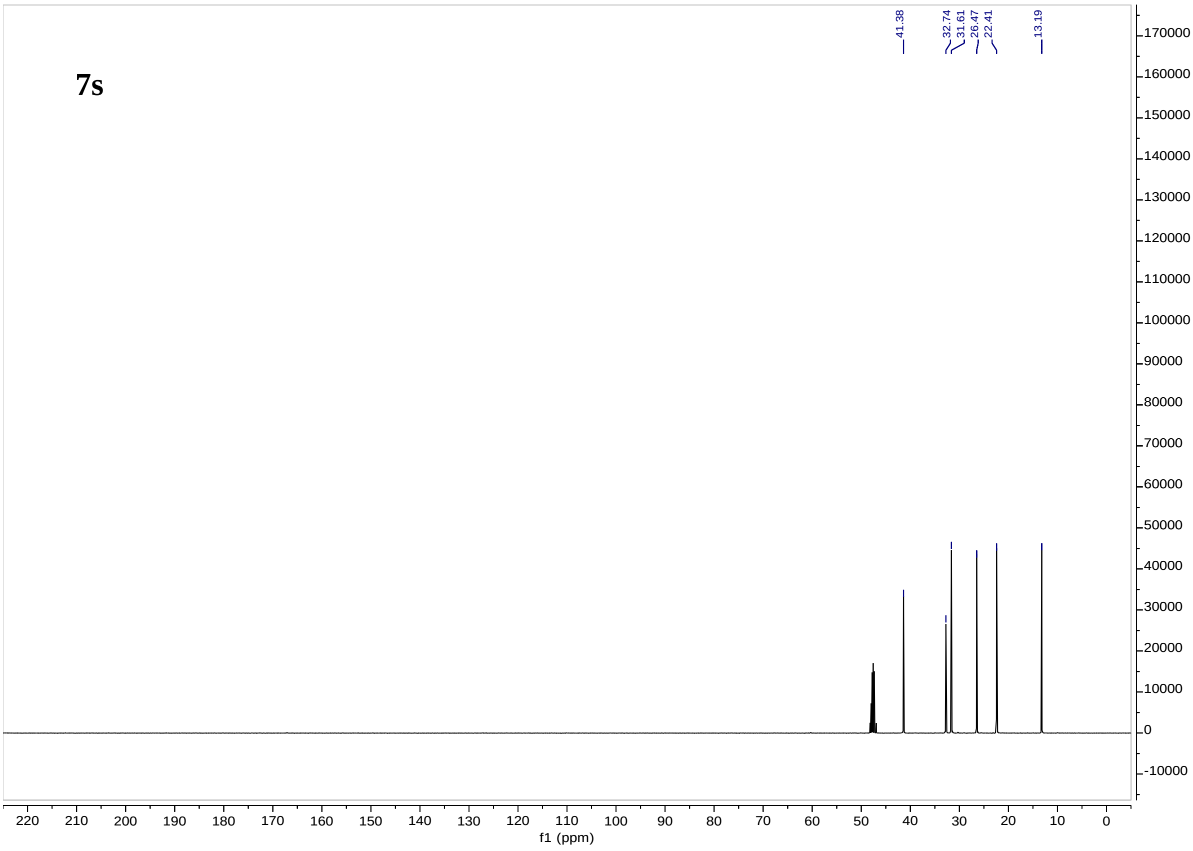

41.38
32.74
31.61
26.47
22.41
13.19
f1 (ppm)

7s

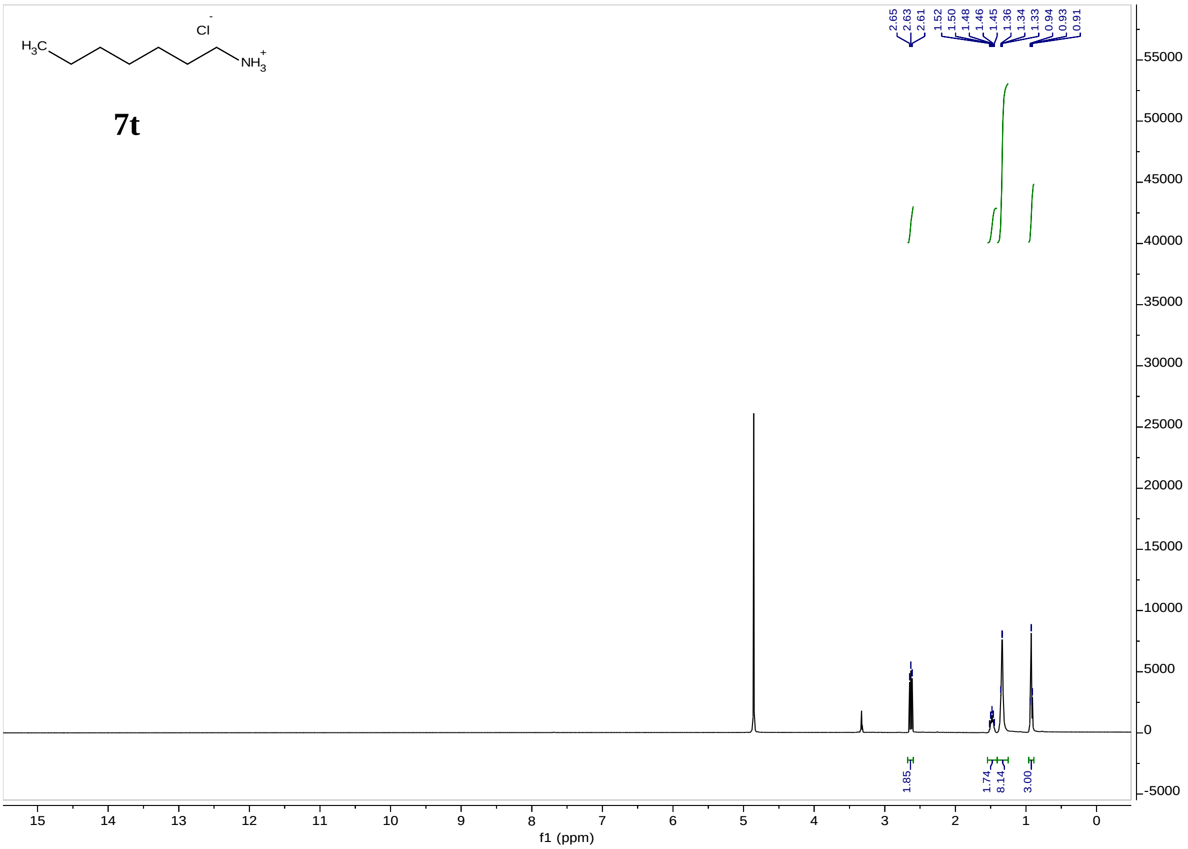

H3C
Cl-
NH3+
7t
2.65
2.63
2.61
1.52
1.50
1.48
1.46
1.45
1.36
1.34
1.33
0.94
0.93
0.91
1.85
1.74
8.14
3.00
55000
50000
45000
40000
35000
30000
25000
20000
15000
10000
5000
0
-5000
15 14 13 12 11 10 9 8 7 6 5 4 3 2 1 0
f1 (ppm)

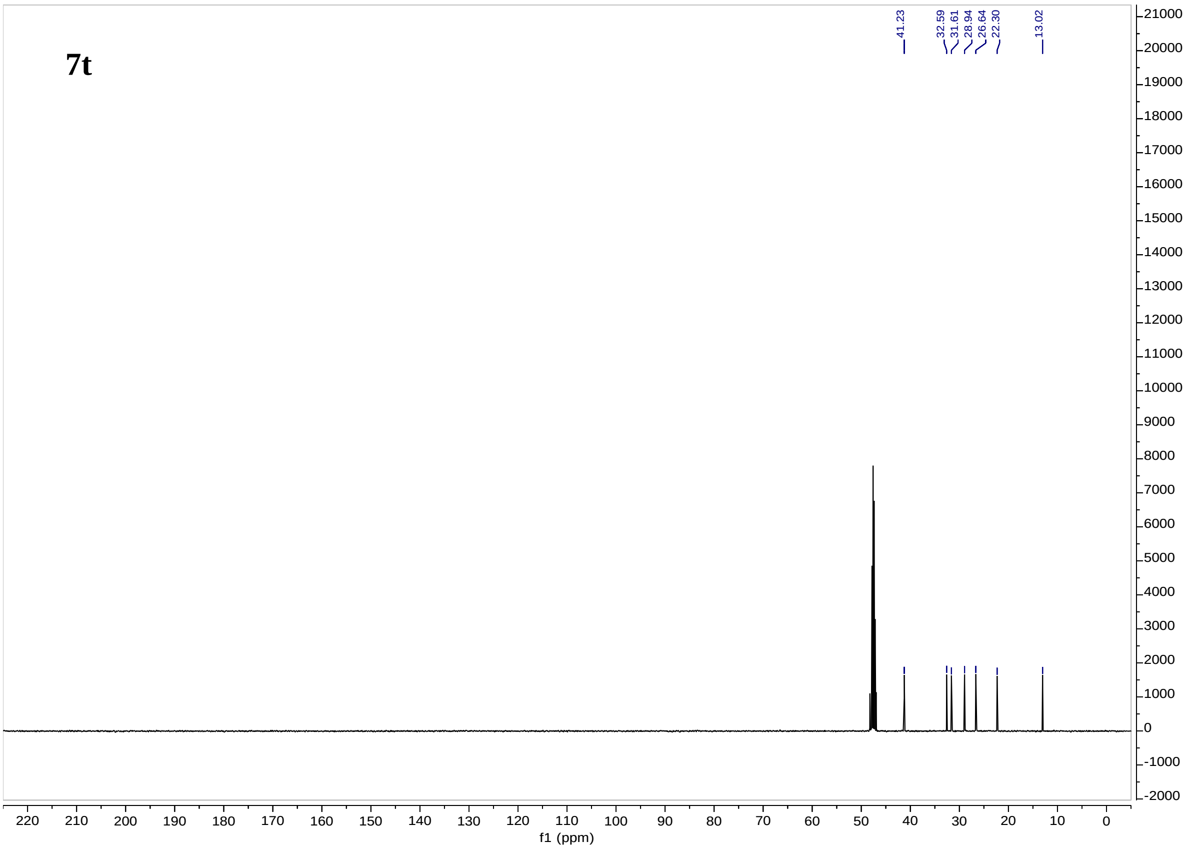

7t
41.23
32.59
31.61
28.94
26.64
22.30
13.02
21000
20000
19000
18000
17000
16000
15000
14000
13000
12000
11000
10000
9000
8000
7000
6000
5000
4000
3000
2000
1000
0
-1000
-2000
220 210 200 190 180 170 160 150 140 130 120 110 100 90 80 70 60 50 40 30 20 10 0
f1 (ppm)

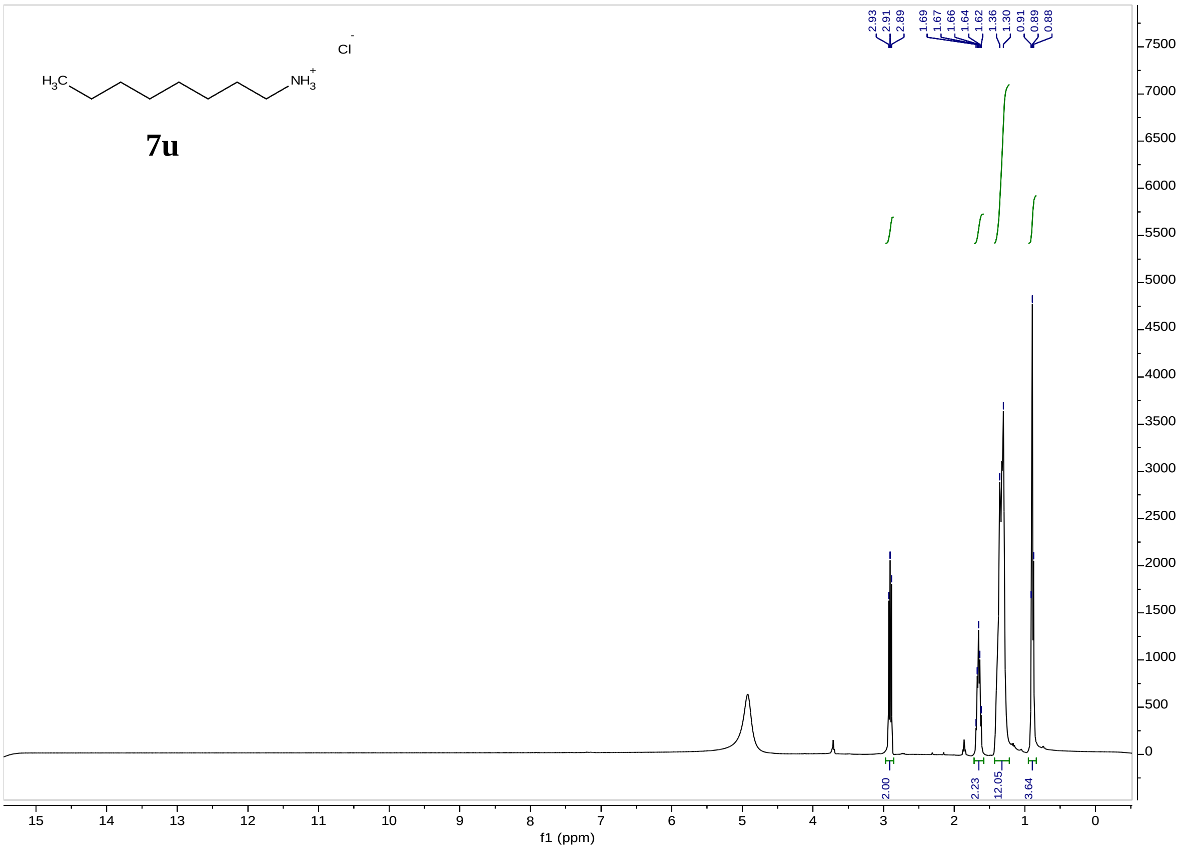
Cl-
H3C
NH3+
7u
2.93
2.91
2.89
1.69
1.67
1.66
1.64
1.62
1.36
1.30
0.91
0.89
0.88
2.00
2.23
12.05
3.64
f1 (ppm)

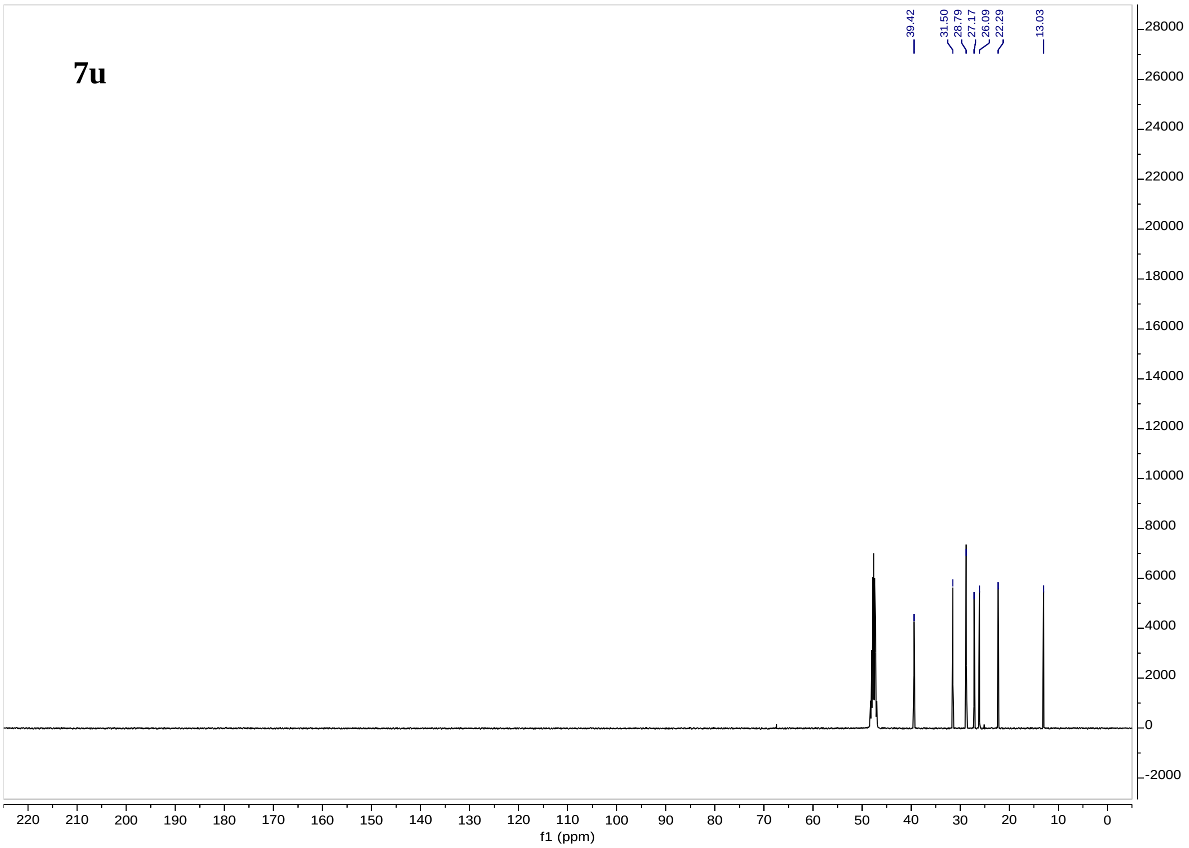
7u
39.42
31.50
28.79
27.17
26.09
22.29
13.03
f1 (ppm)

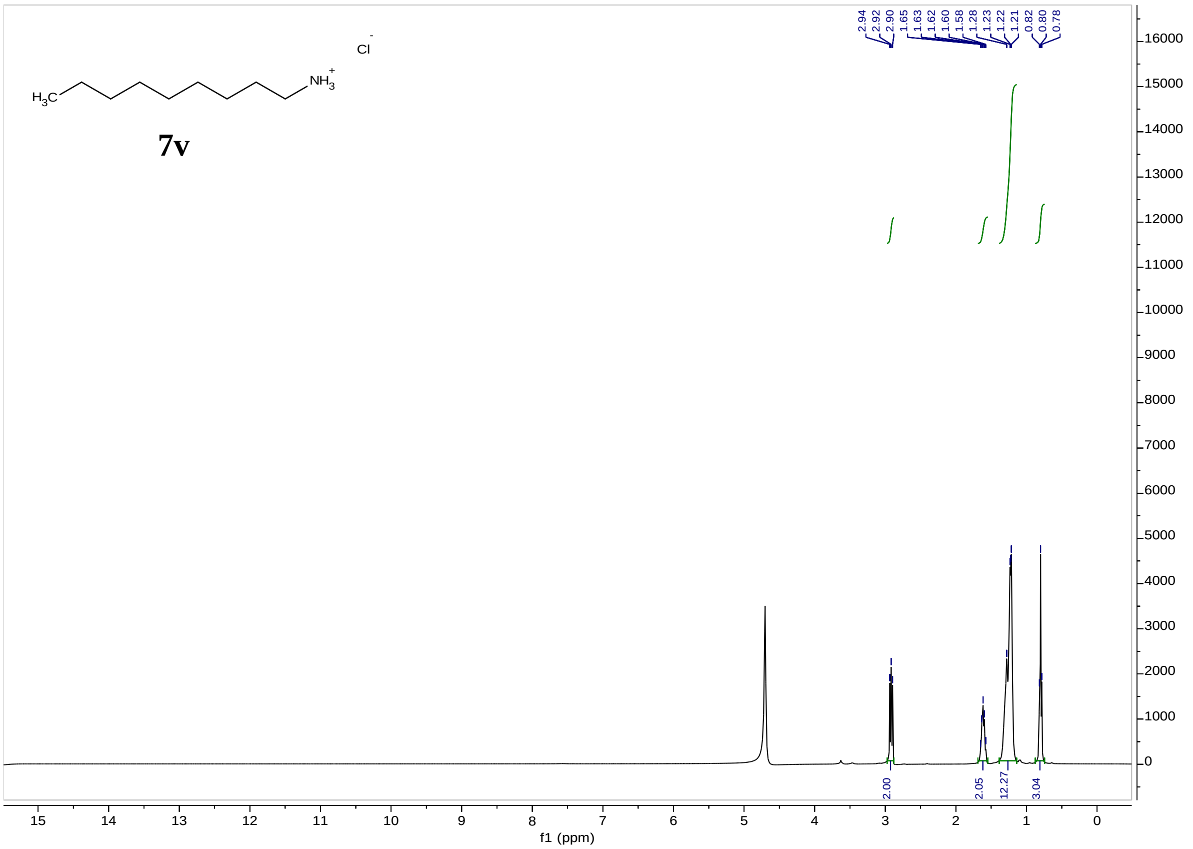
7v
2.94
2.92
2.90
1.65
1.63
1.62
1.60
1.58
1.28
1.23
1.22
1.21
0.82
0.80
0.78
2.00
2.05
12.27
3.04
f1 (ppm)

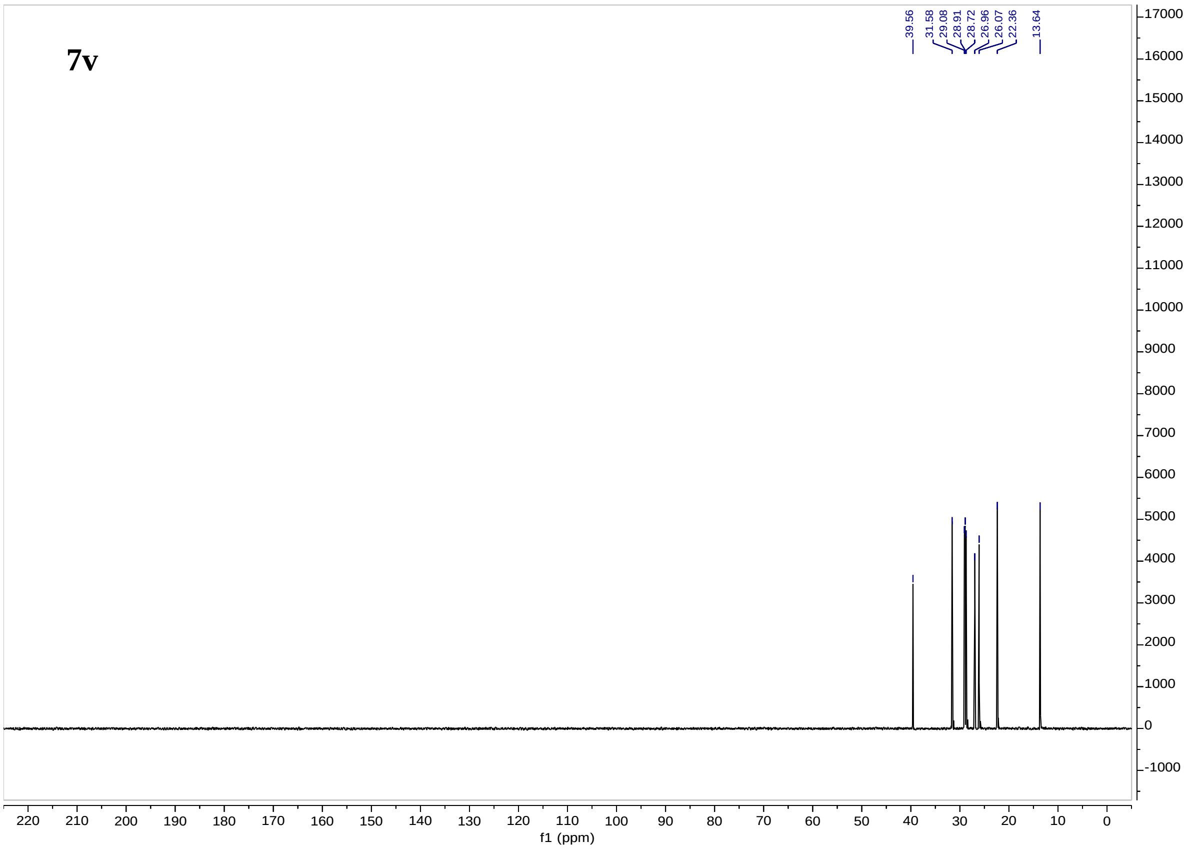
7v
39.56
31.58
29.08
28.91
28.72
26.96
26.07
22.36
13.64
f1 (ppm)

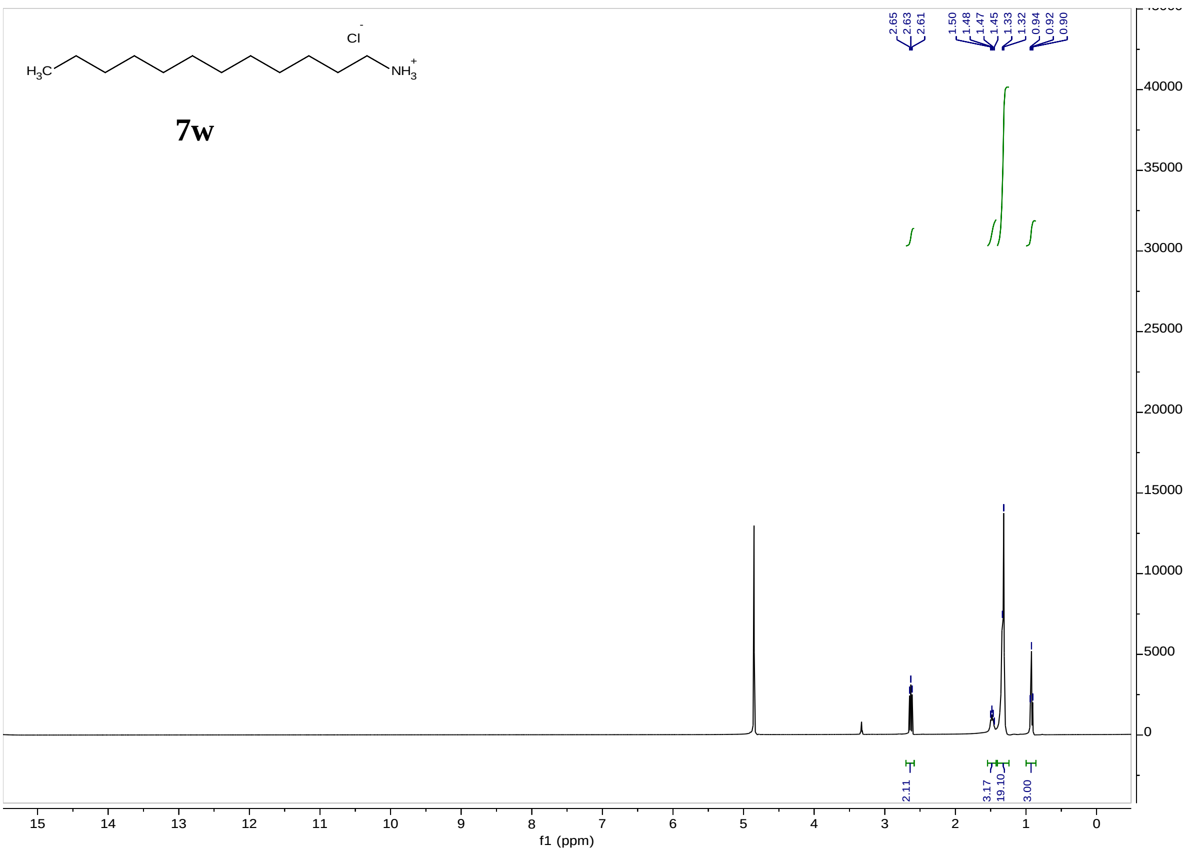

Cl
H3C
NH3
2.65
2.63
2.61
1.50
1.48
1.47
1.45
1.33
1.32
0.94
0.92
0.90
2.11
3.17
19.10
3.00
f1 (ppm)

7w

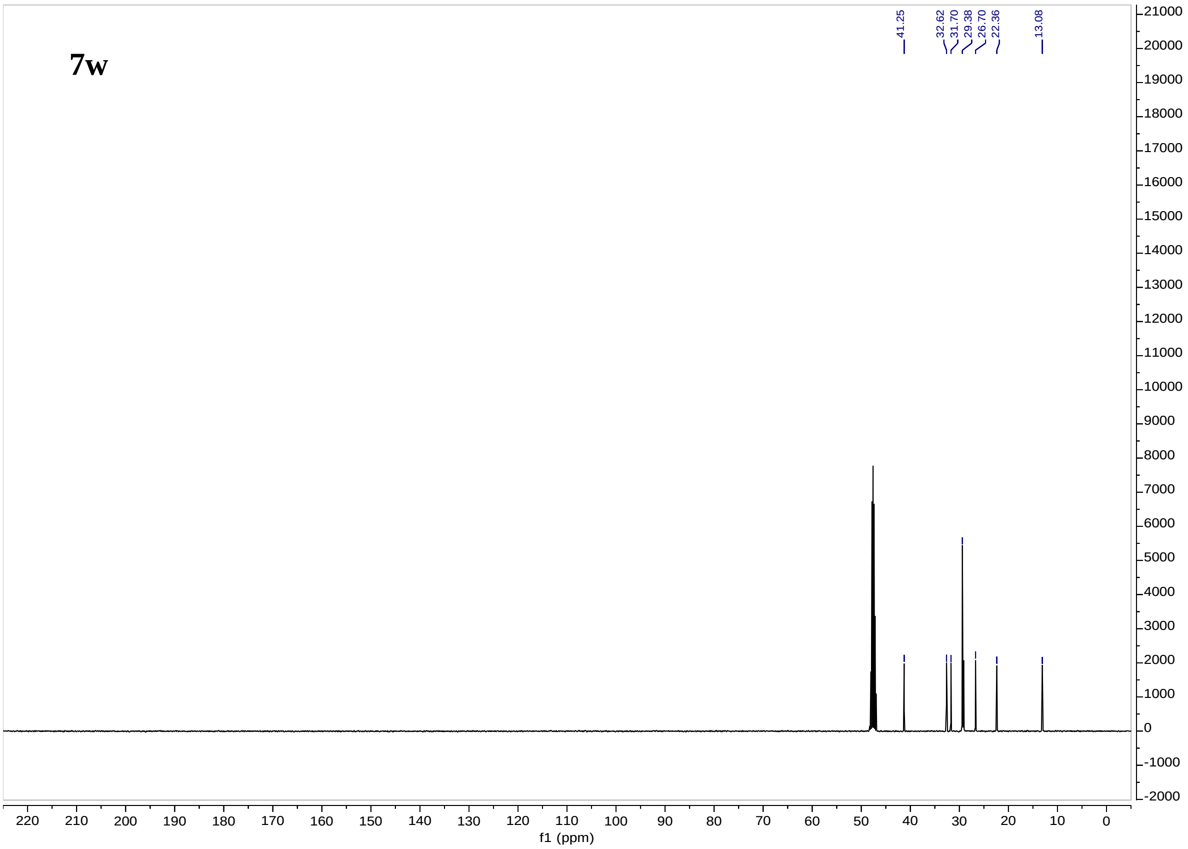

41.25
32.62
31.70
29.38
26.70
22.36
13.08
f1 (ppm)

7w
